# Exploring Rotational Diffusion with Plasmonic Coupling

**DOI:** 10.1021/acsphotonics.3c01482

**Published:** 2024-02-06

**Authors:** Nasrin Asgari, Martin Dieter Baaske, Jacco Ton, Michel Orrit

**Affiliations:** † Huygens-Kamerlingh Onnes Laboratory, Leiden University, Postbus 9504, 2300 RA Leiden, The Netherlands; ‡ Max Planck Institute of Biophysics, Max-von-Laue-Str. 3, 60438 Frankfurt am Main, Germany

**Keywords:** rotational diffusion, gold nanorods, plasmonic coupling, plasmonic ruler, rotational correlation

## Abstract

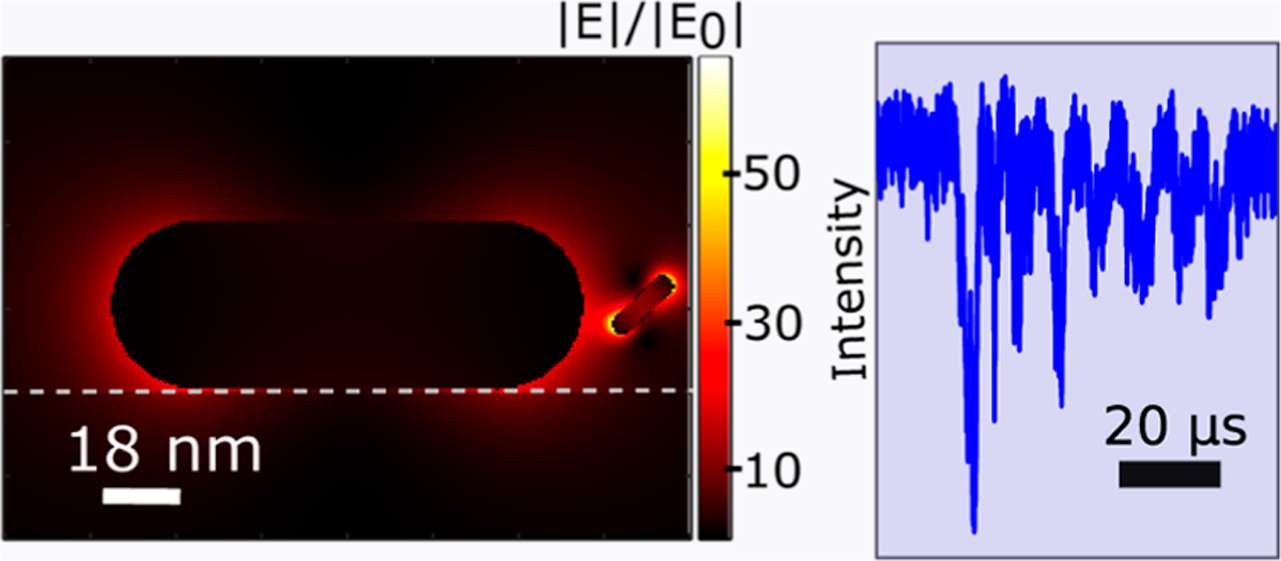

Measuring the orientation dynamics of nanoparticles and nonfluorescent molecules in real time with optical methods is still a challenge in nanoscience and biochemistry. Here, we examine optoplasmonic sensing taking the rotational diffusion of plasmonic nanorods as an experimental model. Our detection method is based on monitoring the dark-field scattering of a relatively large sensor gold nanorod (GNR) (40 nm in diameter and 112 nm in length) as smaller plasmonic nanorods cross its near field. We observe the rotational motion of single small gold nanorods (three samples with about 5 nm in diameter and 15.5, 19.1, and 24.6 nm in length) in real time with a time resolution around 50 ns. Plasmonic coupling enhances the signal of the diffusing gold nanorods, which are 1 order of magnitude smaller in volume (about 300 nm^3^) than those used in our previous rotational diffusion experiments. We find a better angular sensitivity with plasmonic coupling in comparison to the free diffusion in the confocal volume. Yet, the angle sensitivity we find with plasmonic coupling is reduced compared to the sensitivity expected from simulations at fixed positions due to the simultaneous translational and rotational diffusion of the small nanorods. To get a reliable plasmonic sensor with the full angular sensitivity, it will be necessary to construct a plasmonic assembly with positions and orientations nearly fixed around the optimum geometry.

## Introduction

Rotational diffusion^1^ of biomolecules and nanoparticles in fluids is an important property which has often been ignored because of its very short time scales, in the range of nanoseconds to microseconds.^2,3^ Yet, it scales with the volume of the particle (instead of its diameter for translational diffusion) and is therefore more sensitive to a particle’s size, shape, or conformation than translational diffusion.^4^ Recently, we published a study of rotational diffusion of gold nanoparticles in a confocal microscope,^5^ where the diffusers were far from any surface and were isolated from one another. As the photon budgets offered by typical fluorescence are too low to directly monitor rotational dynamics occurring on times shorter than microseconds, we used a fast scattering-based method. In the present work, we investigate rotational diffusion of small diffusing gold nanorods,^6,7^ while they cross the near field of a larger, immobilized GNR, acting as a sensor.^8^ The plasmonic coupling^9−12^ taking place between the sensor nanorod and the diffusing nanorod has the advantage of increasing the sensitivity with respect to a direct confocal detection,^5^ and of enabling the detection of smaller nanoparticles, which may be less perturbing for biomolecules, for example. Plasmonic coupling, however, has the disadvantage of further shortening the interaction time as the extent of the near field is much reduced in comparison to that of the confocal volume.

Optoplasmonic detection exploits the large coupling of plasmonic metallic nanoparticles to light, arising from their sharp and intense plasmon resonances. In contrast to fluorescence, metal nanoparticles do not bleach nor blink even at high excitation intensities. Scattered light provides a steady and strong flux of photons, therefore high detection bandwidths in the GHz range, corresponding to nanosecond time resolutions. Early applications of optoplasmonic systems have been to monitoring of distance changes.^13^ In such plasmonic rulers, the scattering spectrum and intensity are modified in a measurable way when the respective distance between plasmonic nanoparticles is altered by a biomolecular conformation change.^14,15^ Here, we focus on gold nanorods (GNRs)^7^ which are sensitive to small refractive index changes in their near field. GNRs present highly anisotropic optical properties.^7,16−21^ Their scattering signals are highly sensitive to their orientation, which enables rotational diffusion studies.^5^ Single GNRs can work as optoplasmonic sensors detecting single-protein binding events, as done in 2012 in demonstration experiments by Zijlstra et al.^22^ and Ament et al.^23^ and more recently by Celiksoy et al.^24^ According to Baaske et al., GNR-based sensors can provide time resolutions down to tens of nanoseconds, as demonstrated with freely diffusing single nanoparticles^8^ and proteins (hemoglobin and glucose oxidase).^5^ Detecting the much subtler changes of refractivity due to reorientation of an anisotropic biomolecule would be much more challenging. Indeed, simulations indicate that this rotational signal would be three times harder to detect than the molecule’s translational diffusion. Plasmonic coupling simulations suggest that it is roughly 1000 times easier to detect the rotation of a plasmonic nanorod of similar volume (see Section S1.1). Therefore, we focus here on plasmonic coupling to a sensor GNR as a benchmark in the exploration of single-protein rotational diffusion. Compared to a plasmonic ruler made out of two spheres,^25^ a dimer of GNRs is highly sensitive to angles and therefore to rotational diffusion.^26^ Resonantly coupled pairs of plasmonic nanorods further enhance signal and sensitivity. As it is very challenging to build a construct with two nanorods at fixed positions and with an angular degree of freedom, we focus on a much simpler system consisting of freely diffusing gold nanorods crossing the near field of a larger, immobilized sensor GNR.

**Figure 1:**
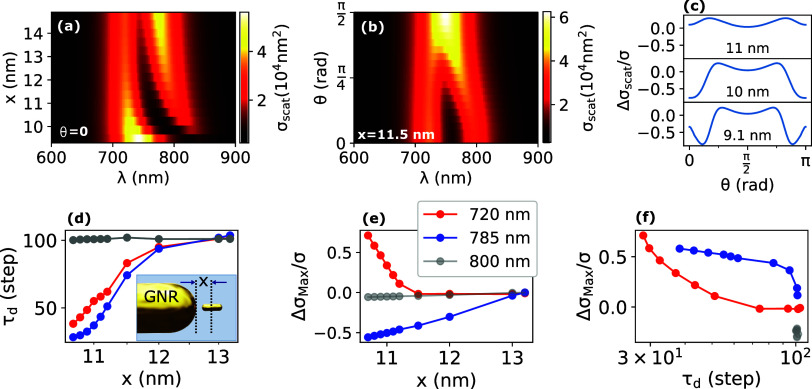
Simulation results. (a) Scattering cross section σ_scat_ of a sensor GNR (112 × 40 nm^2^) and a diffusing rod (18 × 5 nm^2^) placed near the tip of the sensor GNR [schematic shown in (d)] with fixed angle θ = 0 and distance sweep. Note how the splitting due to plasmonic coupling decreases at large distances (*x*). (b) Scattering cross section σ_scat_ of the sensor-diffusing-rod system with fixed *x* = 11.5 nm center-to-surface distance as a function of diffusing-rod orientation between 0 to π/2. Again, the splitting decreases steeply with the angle. (c) Angle dependence of the scattering cross section σ_scat_ of the sensor-diffusing-rod system for three distances of the surface to center (9.1, 10, and 11 nm; gaps 0.1, 1, and 2 nm) and for λ = 785 nm. (d) Inset: scheme of the sensor GNR and the diffusing rod with surface to center distance *x*. τ_d_ versus distance *x* for three different wavelengths: 720 nm orange, 785 nm blue, and 800 nm gray. τ_d_ has been deduced from an orientation random walk of the diffusing rod for each fixed surface to center distance *x*. (e) Normalized deviation of the scattering cross section (Δσ_scat_/σ) of the sensor-diffusing-rod system versus the distance *x* for three different wavelengths. (f) Correlation plot of the Δσ_scat_/σ of the sensor-diffusing-rod system with the simulated rotational correlation time for the same wavelengths as in (e). Large fluctuations are correlated with shorter diffusion times.

**Figure 2:**
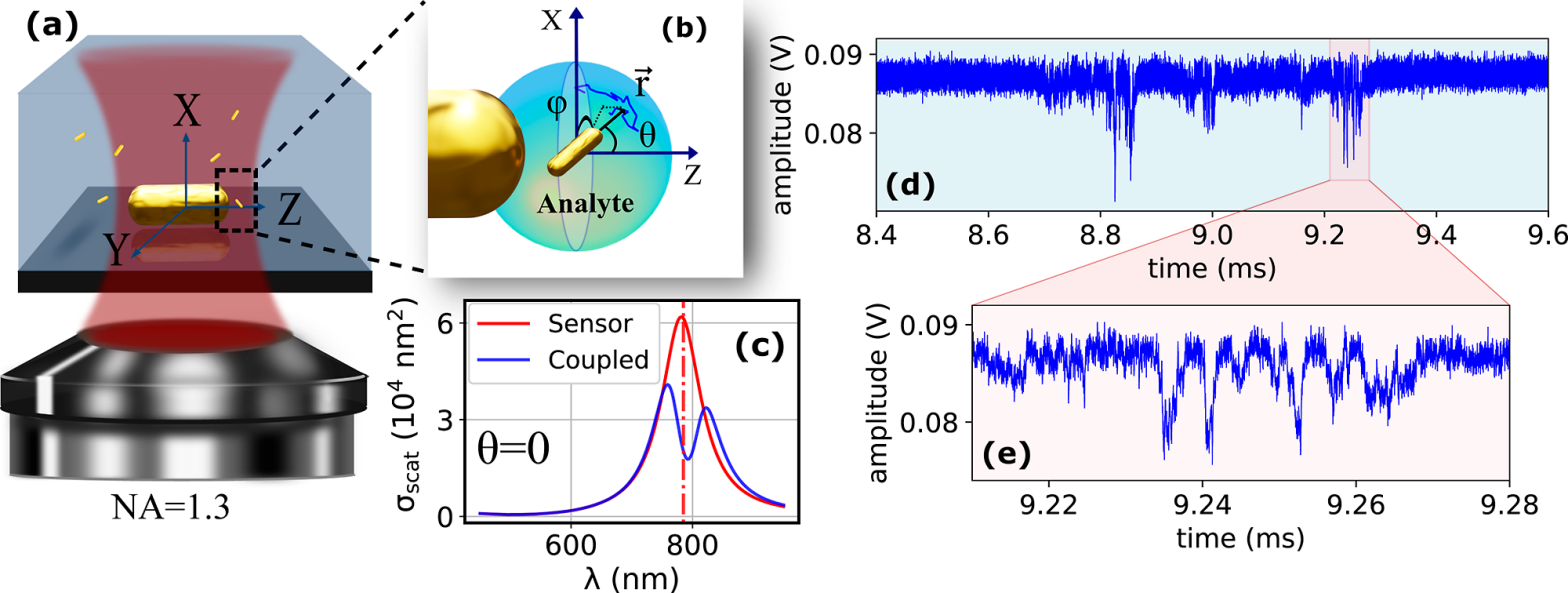
Schematic representation of the sensor-diffusing-rod system. (a) Sensor GNR 112 × 40 nm^2^ is immobilized on a glass coverslip located at the focus of the oil immersion objective of a confocal microscope. (b) Zoomed-in schematic of the sensor GNR with a randomly diffusing rod with polar θ and azimuthal ϕ angles. For the calculations, we have assumed the center of the diffusing rod to lie on the axis of the GNR. (c) Simulated scattering cross section of the sensor GNR 112 × 40 nm^2^ (red) and of the coupled dimer of sensor-diffusing-rod with aligned axes and a gap of 5 nm from surface-to-surface (dark blue), for light polarized linearly along the sensor’s long axis. The diffusing rod in this case is a 19 × 5 nm^2^ gold nanorod. Note the spectacular change in scattering spectrum caused by the diffusing rod which is 360 times smaller in volume than the sensor GNR, due to plasmonic coupling between the rods. (d,e) Measured scattering time trace of the sensor GNR showing clear perturbations (events) between 8.6 and 9.4 ms assigned to the diffusion of a single diffusing rod (19.1 × 5.9 nm^2^) through the near field. The strong signal is due to transient plasmonic coupling. The fast fluctuations correspond to the rotational diffusion, whereas the extended event is assigned to translation Brownian diffusion of a rod through the sensor’s near field. The time resolution in our measurement is 50 ns, which enables the detection of sub-bursts as fast as 100 ns in this case (see Section S5).

**Figure 3:**
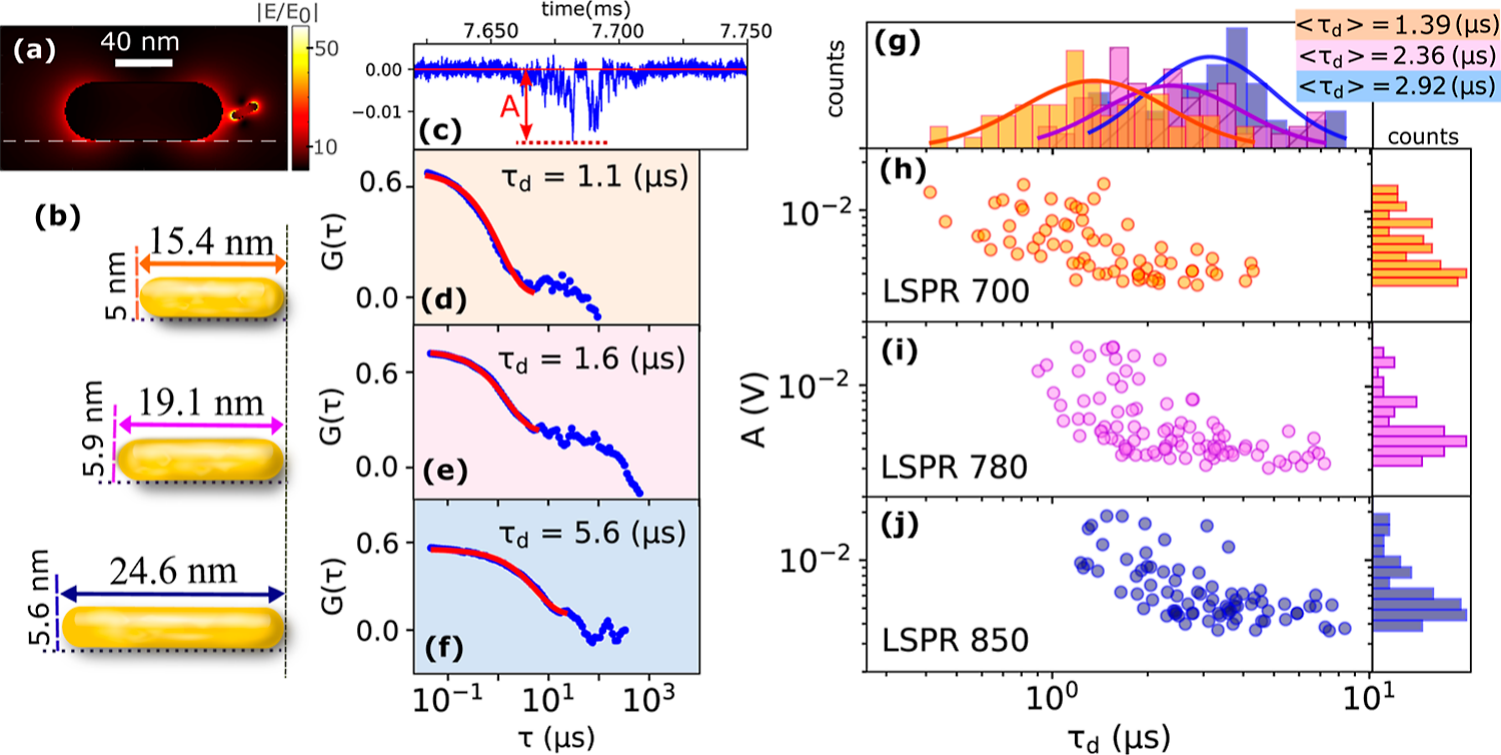
(a) Map of the optical electric field around a sensor GNR 112 × 40 nm^2^ on a glass substrate in the presence of a diffusing rod (19.1 × 5.5 nm^2^) in the near field. Note the very strong field enhancement in the vicinity of the diffusing rod. (b) Sketch of the three rod samples with different aspect ratios, all of them are around 5 nm in diameter (see Section S3 for SEM) with 15.4, 19.1, and 24.6 nm as the average length. (c) Example of an event caused by a 19.1 × 5 nm^2^ diffusing rod with maximum amplitude of *A*. (d–f) Examples of the single event’s autocorrelation measured with the same sensor GNR for each rod samples which show how we have extracted the decay times. The red curves are single-exponential fits with respective decay times τ_d_ = 1.1 μs, τ_d_ = 1.6 μs, and τ_d_ = 5.6 μs. (g) Probability histograms of decay times of the fast component for the near-field measurements performed for three rod samples with the same sensor GNR. The histograms are normalized to the number of events which are 68, 95, and 81 events, respectively. The histograms are fitted with log–normal distributions, with mean values ⟨τ_d_⟩ listed. (h–j) Scatter plots of the maximum amplitude *A* of the scattering signal versus the decay time τ_d_ of the correlation for a number of events and the three samples of diffusing rods, all measured with the same sensor GNR.

**Figure 4:**
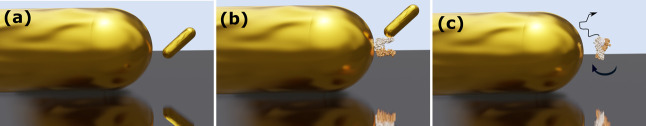
Few examples of optoplasmonic detection schemes based on a single sensor GNR. (a) Free small gold nanorods translationally diffusing and tumbling in the near field of the sensor GNR. (b) This detection may be even more sensitive if the diffusing nanorod is attached to a fixed position near the tip of the GNR by a biomolecule (Hsp90) and will be able to detect the conformational changes of the molecule. (c) Refractive-index-based detection of a single protein freely diffusing through the near-field zone of the sensor GNR.

We also performed simulations of optoplasmonic sensors of rotational diffusion on a coupled system comprising a large sensor GNR and a smaller nanorod freely diffusing in angle but at a fixed position. To enhance coupling, the two particles are chosen such that they are close to mutual resonance. The small diffusing nanorod (5 nm in diameter) is placed in the proximity of a larger sensor GNR (112 × 40 nm^2^), and we monitor the sensor’s dark-field scattering (neglecting the weak reflection of the glass–water interface) with a high time resolution. We estimate the angle sensitivity and signal-to-noise ratio in a given bandwidth, in view of future applications. Plasmonic confinement enhances the electromagnetic field acting on the small diffusing nanorod, and plasmonic coupling between the sensor and the diffuser has a dramatic influence on the scattering signal (see simulations in Figure S2). The high sensitivity and temporal resolution of plasmonic scattering enable our experimental study of the rotational diffusion of small diffusing nanorods, which diffuse translationally as well as rotationally. From measured time traces, we estimate the corresponding angle sensitivity. This factor enables us to discuss the feasibility of measuring the orientational dynamics of an unlabeled single molecule through this scattering method.

## Simulations

To understand the coupling effect, we have performed simulations of the scattering cross section for various angles and distances between two coupled nanorods, a sensor GNR (112 × 40 nm^2^) and a small rod (hereafter called diffusing rod) (18 × 5 nm^2^). We first calculate the scattering cross section σ_scat_ of the sensor-diffusing-rod system when the sensor’s and diffusing-rod’s axes are parallel (θ = 0) and the diffusing rod is moved by a variable distance *x* between its center and the surface of the sensor GNR (thus, the gap between the two metal surfaces is *x* − 9 nm). Figure 1a shows a splitting in the scattering spectrum when the diffusing rod is brought close to the sensor. The splitting between the two hybridized modes increases upon a decrease of the distance.^27^ Note that our simulation solves Maxwell’s equations by a boundary element method (BEM).^28^ Errors due to the finite mesh size as well as quantum phenomena, which appear for extremely short distances between gold nanoparticles, have not been considered in these simulations. Such errors are expected to affect mostly the simulation for *x* = 9.1 nm.

To investigate the effect of the angle on the splitting (see Section S1.3), we now keep the distance constant at *x* = 11.5 nm (gap 2.5 nm) and sweep the angle from 0 to π/2. Figure 1b shows two branches, which display maximum splitting at θ = 0 and no coupling at θ = π/2. The influence of diffusing-rod rotation on the scattering is very pronounced in the wavelength range (720–785 nm). Furthermore, we plot the relative angular variation of the sensor GNR’s scattering cross section, Δσ_scat_/σ, as a function of θ for a few distances (9.1, 10, and 11 nm) in Figure 1c. The plots can be fitted with power series of cos^2^θ (see Section S1.5), whereas higher-order contributions decay rapidly with increasing distance and become negligible beyond 30 nm.

A convenient way to study the dynamic behavior of nanoparticles is through the correlation function of the light intensity they scatter.^5,29^ Rotational diffusion’s fast dynamics appears as a short-time scale component in the autocorrelation curve. To measure the rotational correlation function, the diffuser should have anisotropic optical properties like a gold nanorod. To estimate the effect of the distance on the rotational correlation time τ_d_, we simulated an orientational random walk^5^ of the small rod at a fixed position with respect to the sensor GNR (see Section S1). Calculated autocorrelation functions show a clear single-exponential decay (Figure S1c) with decay constant τ_d_ (expressed here in units of simulation steps instead of time; one step corresponds to about 20 ns). At both wavelengths 720 and 785 nm (as examples), the rotational correlation time decreases markedly with distance. The faster decay at short distances arises from the stronger angle dependence of the scattering, i.e., from higher powers of cos^2^ θ in the scattering cross section (Figure 1c). Smaller distances enhance plasmonic coupling and yield higher deviations Δσ_scat_ of the scattering cross section and stronger scattering signals (especially for 720 nm wavelength). The angle dependence and the distance dependence at close distances are stronger. According to Figure 1c, the angle sensitivity is maximum at around θ = π/8 and close distances (roughly less than *x* = 12 nm, i.e., 3 nm of metal–metal gap).

These plasmonic coupling simulations were carried out with two quasi-resonant rods to maximize coupling effects. As expected, splitting is maximal for small inter-rod distance when the diffusing rod is placed on the longitudinal axis of the sensor GNR and for aligned longitudinal axes (see Figure 1). Figure 1b indicates that at the distance of 11.5 nm, the splitting is maximal for aligned rods. However, the plots of (c) show that the maximum angle sensitivity is obtained with a small misalignment of rods (about 20–30°), essentially corresponding to larger variations of the gap between the rods when the angle of the diffusing rod is varied. Even in this optimal case, and for a gap as small as 1 nm between the two rods (distance 10 nm in Figure 1c), plasmonic coupling merely enhances the angle sensitivity about three to four times compared to the far-field confocal configurations (see Section S9). Nonetheless, plasmonic coupling enables the observation of small nanorods with scattering signal contrasts (more than 10%) exceeding the entire scattering cross section of small NRs by multiple orders of magnitude, thus enabling their detection with high bandwidth. However, plasmonic coupling between sensor and diffusing rod leads to strong intrication of translational and rotational degrees of freedom in the optical scattering signal. In particular, the autocorrelation decay time (Figure 1d) τ_d_ markedly decreases at small distances for 720 and 785 nm wavelengths (schematic in Figure 1d) even though the distance is kept constant in the simulation. We shall come back to this observation in our discussion of the experimental results.

## Experiment

The measurement setup has been discussed earlier^30,31^ and is built around the oil-immersion objective of a confocal microscope (see scheme in Section S2). To measure the fast and weak signals from small diffusing rods, we measure intensity variations in the dark-field scattering by the sensor GNR upon diffusion of rods in its near field. As shown in Figure 2a, the sensor GNR (112 × 40 nm^2^, see the Methods section for details) is immobilized on a glass coverslip in a flow cell. We probe the sensor GNR with linearly polarized light along its long axis and record the scattering time trace with an analyzer in the same orientation.^30,31^ Suspensions of diffusing rods with 5.0 ± 0.5 nm diameter and three different lengths (15.5, 19.2, and 24.6 nm, see Section S3) were injected into the flow cell consecutively, keeping the same sensor GNR. We chose the incident laser power (38 μW) low enough to avoid any reshaping of the sensor GNR during our measurements, which could extend up to hours (see Section S4). Moreover, according to absorption and heat conduction simulations, the changes of temperature and of water viscosity around the sensor GNR were found to be negligible (see Section S4.1). Therefore, we do not expect thermophoresis to play any significant role in our measurement. Furthermore, optical torques and the associated laser-induced rotations are not significant compared to thermal energy at the incident power used, even taking into account enhancement by the sensor rod.^19,20^

## Results and Discussion

We have studied the coupled system of an immobilized sensor GNR and freely diffusing small gold nanorods. When one of those diffusing gold nanorods approaches the sensor GNR, their near fields overlap and their plasmonic dipole modes strongly couple with each other, which modifies the plasmonic resonance energies and the scattering cross section of the whole system.^26,32^ The plasmon coupling between two gold nanorods and the angle dependence of the coupled modes are explained qualitatively by a modified version of the Simpson-Peterson approximation.^11,26,33−35^ In a gold nanorod dimer, the two plasmonic dipole modes of individual nanorods couple together and yield two eigenmodes at different energies (antibonding and bonding modes) causing the mode splitting appearing in Figure 2c.^9,36,37^ As we probe the sensor GNR with a specific wavelength, the scattered intensity will generally be significantly altered by coupling with the diffusing rod. In the case of Figure 2c, and for a wavelength between 770 and 810 nm, the disturbance lowers the scattering intensity. The optical properties of this coupled system at a given wavelength depend on multiple parameters: the angles of the nanorods with respect to each other, the position of the diffusing rod with respect to the sensor, in particular the gap between their tip surfaces.^26,32^ Therefore, this coupled system provides us with an efficient sensing mechanism for displacements and reorientations of the diffusing rod.

The detection in our method is based on the changes of the intensity scattered by the sensor at a specific wavelength due to the transient plasmonic coupling with a diffusing rod. Figure 2d shows a scattering time trace of the sensor GNR. At around 8.7 ms, some fast fluctuations of the intensity appear which we assign to plasmonic interaction with an individual diffusing rod (in this case, a 19.1 × 5.9 nm^2^ gold nanorod). In Figure 2d, we see a group of fast fluctuations starting at 8.7 ms and lasting until around 9.4 ms, which we hereafter name an event. At the highest concentration of the diffusing rods used in our measurement, we find that events account for ≤1% of the entire recording time. Therefore, the probability of detecting more than one diffusing rod in an event (about 1%) is negligible. Inside the event, as the zoomed-in trace (e) shows, the scattered intensity deviates from the unperturbed intensity, with strong and fast fluctuations. We assign those fast fluctuations at least in part to orientation changes of the diffusing rod with respect to the sensor GNR’s long axis. Another source of fluctuations are position changes of the diffusing rod due to translational diffusion with respect to the immobilized sensor GNR. Such positional changes can affect the coupling and consequently the optical properties of the coupled rods. As the orientation changes occur faster than the translational ones, we have ignored the effect of translational diffusion on the fast fluctuations. Therefore, we assign the repeated bursts between 8.7 and 9.6 ms to exploration of different areas of the sensor’s near field by the diffusing rod, which yields various strengths of the coupling. It seems natural to assign the duration of the whole event to the time spent by the diffusing rod in the sensor’s near field before translational diffusion removes it from this area. Therefore, contrary to enhanced fluorescence measurements^38,39^ where most bright events appear isolated and point-like, plasmonic coupling in our experiments appears to give rise to much more complex patterns of bursts including sub-bursts.

To search for such events, we compute the autocorrelation *G*(τ) of the intensity time trace on 10 μs sliding windows. Transient plasmonic interaction causes strong contrast in the autocorrelation color map (see the color map of the autocorrelation shown in Section S6), which is more sensitive to weak events than the time trace, and enables us to quickly focus on the interesting events including weaker ones (see more examples in Section S6). In comparison to our previous measurements (which resolved the rotational diffusion of nanorods in the microscope’s confocal volume^5,29^), the diffusing rod will experience a strongly inhomogeneous distribution of the electric near field and a broad range of field strengths as it diffuses around the sensor GNR. As a result, it can be difficult to separate rotational from translational components for some events in the autocorrelation of the scattered intensity, or to define an effective translational diffusion time τ_*t*_ = *r*^2^/(4*D*_t_) (where *D*_t_ is the translational diffusion coefficient and *r* is a typical dimension of the detection zone around the sensor GNR) as these may depend on how the diffusing rod approaches the sensor GNR (we have neglected any damping due to the wall effect, see Section S7).

For each event in the raw time traces, we estimate its starting and ending times by thresholding the autocorrelation map at contrast larger than 0.18. We then compute the intensity autocorrelation *G*(τ) in this time interval. For most of the individual events, we found two clearly distinct relaxation components. Some examples are shown in Figure 3d–f. The shorter component corresponds to rotational diffusion on times on the order of 1 μs. We therefore assign this shorter component to rotational diffusion and the longer component (with a decay time around 100 μs) to translational diffusion. Based on the translational diffusion coefficient of the diffusing rod close to a surface (see Section S7), we deduce a size *r* of this zone of about 90 nm. Indeed, this long correlation time appears to be clearly associated with the repeated bursts of diffusion, as shown in Figures 2d and 3c and in many of the time traces shown in Supporting Information. A possible explanation of this difference might be an attractive potential between the sensor rod and the diffusing rod, which would lead to an enhanced concentration of the diffusing rods in the vicinity of the sensor and to repeated exploration of the near field if the trapping distance is larger than the near-field extent.

As shown in the examples of Figure 3d–f which are the single event’s autocorrelations (related to the highlighted events in the scattering time trace of Figures S16, S14, and S17, respectively), the fast component is well fitted by a single exponential with a specific decay time τ_d_. To support our assignment of this fast component to rotational diffusion of the diffusing rod, we have measured autocorrelations of the scattered intensity when the diffusers are 10 nm gold nanospheres (see Figure S19), as done for 5 nm nanospheres.^8^ Due to the negligible anisotropy of the nanospheres, and the weak plasmonic coupling between a nanosphere (plasmon resonance at 520 nm) and the sensor GNR (plasmon resonance at 750 nm), we do not expect any rotational diffusion component in the autocorrelation of the traces. Indeed, the autocorrelation function of the trace presented in Figure S19 does not show any short-lived component, contrary to Figure 3d–f.

Furthermore, to study the rotational diffusion of the rods, we have repeated the previous measurements with the same sensor GNR and three samples of diffusing rods with different aspect ratios, consecutively. The diffusers are GNRs with diameter of 5.0 ± 0.5 nm and different lengths (see Figure 3b and Section S3). The electrolyte concentration has been adjusted to 2.5 mM NaCl. After fitting a single-exponential function to the fast component of the autocorrelation of each event (red curve in Figure 3d–f), we extract the decay times τ_d_ and plot normalized histograms for each diffusing-rod sample in Figure 3g. We fit log–normal distributions to the histograms and calculate the mean value of the decay times ⟨τ_d_⟩, which will give us access to the rotational diffusion constant of each rod.

From the histograms of rotational correlation decay times in Figure 3g, we see a clear variation of the inverse rotational diffusion constants (i.e., rotational diffusion times) with the size of the diffusing rod. As expected, the rotational diffusion time increases with the volume of the diffusing rod. The width of these histograms is due to the distribution of sizes and shapes of individual diffusing rods, in addition to the stochastic character of each event.

As a control experiment, we repeated the same measurements without the sensor GNR in the confocal volume,^5^ on the same diffusing rod samples (see Section S8). We observed a slow component in the correlation of the time trace with lower amplitude (Figure S21) in comparison to the near-field measurement. By comparing the average rotational decay times ⟨τ_d_⟩ in the near-field (Figure 3g) and confocal (Figure S22a) histograms, we observe in general a faster decay time in the near field than that in the confocal spot. To be more specific, rotational decay times measured in the near-field were, respectively, 1.5, 21, and 50% faster than the decay times measured under confocal illumination for the nanorod samples with 15.5, 19.1, and 24.6 nm lengths. This observation indicates a higher sensitivity of the near-field measurement to orientation changes compared to the confocal one. Another difference is the broadening of near-field histograms, which we assign to heterogeneity due to the vicinity of the solid surfaces (glass and gold), to a shorter integration time (i.e., shorter event yield less sub-bursts), in addition to the intrinsic polydispersity of the diffusing-rod sample (see Figure S10).

According to Figure 3h–j, the events with higher amplitude *A* have faster decay times τ_d_. We assign this difference to the deeper near-field regions explored by the individual diffusing rod in some of the events. Events with large amplitude correspond to strong plasmonic coupling, when the diffusing rod comes very close to the sensor GNR. In this case, rotational diffusion has very short components corresponding to high-order angle dependence and couples to translational diffusion over very short gap distances of a few nm. In weak events with small amplitudes, the diffusing rod remains relatively far from the sensor, so that the amplitude’s dependence on the orientation of the diffusing rod is not very different from the confocal case. The simulations of Figure 1d–f provide partial explanation for this finding. In the simulation, the diffusing rod is kept at a fixed position but is allowed to rotationally diffuse freely. Indeed, we see that events with large scattering amplitude have considerably shorter diffusion times than weaker events. Translational diffusion during the burst will obviously further enhance this correlation between strong amplitude and short duration.

We have compared the angular sensitivity of the near-field measurement with the confocal one (see Section S9). We found three times better sensitivity for the near field in comparison to the (bright-field) confocal measurement and four times better in comparison to the dark-field confocal measurement. The angle sensitivities found in the experiments fall short of the expectation from simulations of two GNRs at fixed positions and optimized angle (which we hereafter call “tethered system”). Indeed, the plasmonic coupling simulations of Section S9 lead to a maximum angular sensitivity which is about 30 times better than that of our near-field measurements. The better expected sensitivity from the simulation arises from the small and fixed gap between the particles and from their optimal orientation. The sensitivity is reduced in the measurement due to averaging over angles and positions and possibly spectral positions of the diffusing rod’s plasmon resonance. Therefore, building an efficient optoplasmonic tethered system to enable fast measurements of small orientation changes of a probe would require a stable placement of the two particles. This could be realized thanks to proteins or DNA origami constructs^40,41^ maintaining a small gap between the two metal surfaces and the proper angle between them so that the spectrum varies significantly when the angular degree of freedom of interest is activated.

## Conclusions

In the present work, we studied the rotational diffusion of small gold nanorods through plasmonic coupling to another plasmonic particle, a sensor GNR (Figure 4a). We also compared the results with the free diffusion of such gold nanorods through the confocal volume without plasmonic coupling. We found that the sensitivity for angle changes in the plasmonic-coupling configuration is three to four times better compared to the confocal methods. Our simulations suggest that this figure may even be improved to factor of 30 in a more ideal scenario. As a future application to studies of protein conformation changes, optoplasmonic coupling of two gold nanorods can only provide such a significant sensitivity enhancement if a small gap between two plasmonic nanoparticles is changed upon molecular rearrangement (as shown in Figure 4b for Hsp90 protein). This requires careful assembly of the plasmonic particles with the biomolecules of interest. Finally, we can consider the idea of free rotational diffusion of anisotropic dielectric nanoparticles such as protein molecules (Figure 4c). To directly discern 90° differences in molecular alignment would require integration times on the order of ≈60 μs (≈70 ns) using our experimental (simulated) sensitivity as basis. In the ideal simulated situation, rotational diffusion of such nanoparticles in water would be just barely detectable, and the experimental value suggests that diffusion must be somehow slowed down before it can be directly detected by scattering in real time.

## Methods

### Immobilizing Sensor GNRs

The suspension of sensor CTAB-coated GNRs was purchased form Nanopartz as A12-40-750-CTAB-DIH-1-25. According to the supplier, the rods are 112 nm in length and 40 nm in diameter and have a plasmon resonance at 750 nm with concentration of 0.035 nM. The GNR suspension has been diluted, and extra CTAB has been washed away by centrifugation and resuspension in milli-Q water and that solution was sonicated for 30 min. Number 1 coverslips were used as substrates. The coverslips were sonicated in acetone (30 min) and ethanol (30 min) and rinsed with Milli-Q water. Immediately before use, they were ozone-cleaned for 1 h. The sensor GNRs were spin-coated on the cleaned cover glasses with 2000 rpm for 60 s and then the substrates were rinsed with milli-Q water to remove traces of CTAB and unbound GNRs. The samples were ozone-cleaned for 30 min to remove CTAB completely from the GNRs. With this protocol, we have around 45 GNRs per 80 μm^2^ area on the glass.

### Diffusing Rods

The diffusing rods used in this work are citrate-capped with 700, 780, and 850 nm LSPRs and were purchased from Nanopartz as A12-5-700-CIT-DIH-1-25, A12-5-780-CIT-DIH-20-1, and A12-5-850-CIT-DIH-1-25, respectively. The nanorods are, respectively, corresponding to 15.5, 19.1, and 24.6 nm lengths. The concentrations of these rod solutions were 13.8, 245.4, and 8.3 nM, respectively.

The measurements have been carried out by filling the flow cell with a 2.5 mM sodium chloride (NaCl) aqueous solutions containing the three samples of diffusing rods, consecutively. According to the bulk spectrum measurement, the diffusing rods have their average localized surface plasmon resonance (LSPR) at 700, 760, and 880 nm, respectively (see Section S4.2).

### Optical Setup

Here is the list of components that we have used in our setup: Laser: Toptica DL pro 785 nm, APD: Thorlabs APD430A/M (DC-Coupled), Piezo Translator P-561.3CD (Physik Instrumente GmbH & Co KG), Glan-Thompson Polarizer GTH10M-B (Thorlabs), 10:90 Beamsplitter BSN11 (Thorlabs), Tube lens: Olympus Super Wide Tube Lens Unit, White-light source: EQ-99XFC (Energetiq), Spectrometer: QE-65000 (Ocean Optics), and Oscilloscope: LeCroy Wavesurfer 200 MHz

Our detector has a −3 dB bandwidth of 400 MHz, and we have sampled our data after a low-pass filter (190 MHz) with the oscilloscope and a rate of 50 MHz. The response time of the detector and connected electronics is 5 ns. An 8-point median filter has been applied to all the time traces in this work. Therefore, the time resolution in our measurement traces is around 50 ns.

## Supporting Information Available

Simulation; optical setup; diffusing rods; temperature estimation around the sensor GNR; time resolution; more events with different diffusing rods; translational diffusion; confocal monitoring of the rotational diffusion of diffusing rods; and angle sensitivity (PDF)
