# Supplementary material for: Exploring Rotational Diffusion with Plasmonic Coupling
Source: ACS Photonics. 2024 Feb 6;11(2):634–41. doi: 10.1021/acsphotonics.3c01482 (PMC10885195; doi:10.1021/acsphotonics.3c01482)
Supplement: Supplementary file 1 — ph3c01482_si_001.pdf [file ph3c01482_si_001.pdf]

Supporting Information: Exploring Rotational Diffusion With Plasmonic Coupling

Nasrin Asgari,[†] Martin Dieter Baaske,[†,‡] Jacco Ton,[†] and Michel Orrit*,[†]

†*Huygens-Kamerlingh Onnes Laboratory, Leiden University, Postbus 9504, 2300 RA Leiden, The Netherlands*

‡*Max Planck Institute of Biophysics, Max-von-Laue-Str. 3, 60438 Frankfurt am Main, Germany*

E-mail: orrit@physics.leidenuniv.nl

S1 Simulation

Calculations of scattering cross sections σ_{scat} in this work have been done by MNPBEM toolbox in MATLAB.[1]

S1.1 Dielectric rotationally diffusing nanorod

To evaluate the feasibility of measuring the rotational diffusion of dielectric particles or proteins with our method, we perform an orientational random walk simulation (for the details of the simulation look at our previous work[2]). The small nanorod is a $22{\times}5\text{nm}^2$ nanorod which is located at a fixed point near the tip of a sensor GNR $112{\times}40\text{nm}^2$ with $x = 16$ nm distance (surface of the sensor to center of the small nanorod). The small nanorod is freely rotating around the fixed point. The result shows that the scattering cross section of the GNR-rotating-rod system does depend on the orientation of the small nanorod. In the case of a dielectric rotating rod, this dependence will be due to the anisotropic polarizability

of the rotating rod, but the coupling being very small, there will be no visible plasmonic mode splitting, in contrast to the case of the rotating rod (in fig. S2). Moreover, the $\Delta\sigma_{scat}$ for a dielectric rotating rod doesn't change sign because the refractive index changes of the GNR environment can cause only a red shift in the spectrum of the GNR (even at $\pi/2$ angle of the rotating rod. We also have calculated the autocorrelation of the random walk trace shown in fig. S1 (a) for long enough trace (10^6 steps). The calculated autocorrelation, shown in (c) manifests a clear single-exponential decay. The single-exponential fitted function (red) has a decay constant of 100.5 steps. Each step of this particular random walk can be roughly estimated by comparing with the experimental histogram of this nanorod sample shown in fig.4g in the main text.

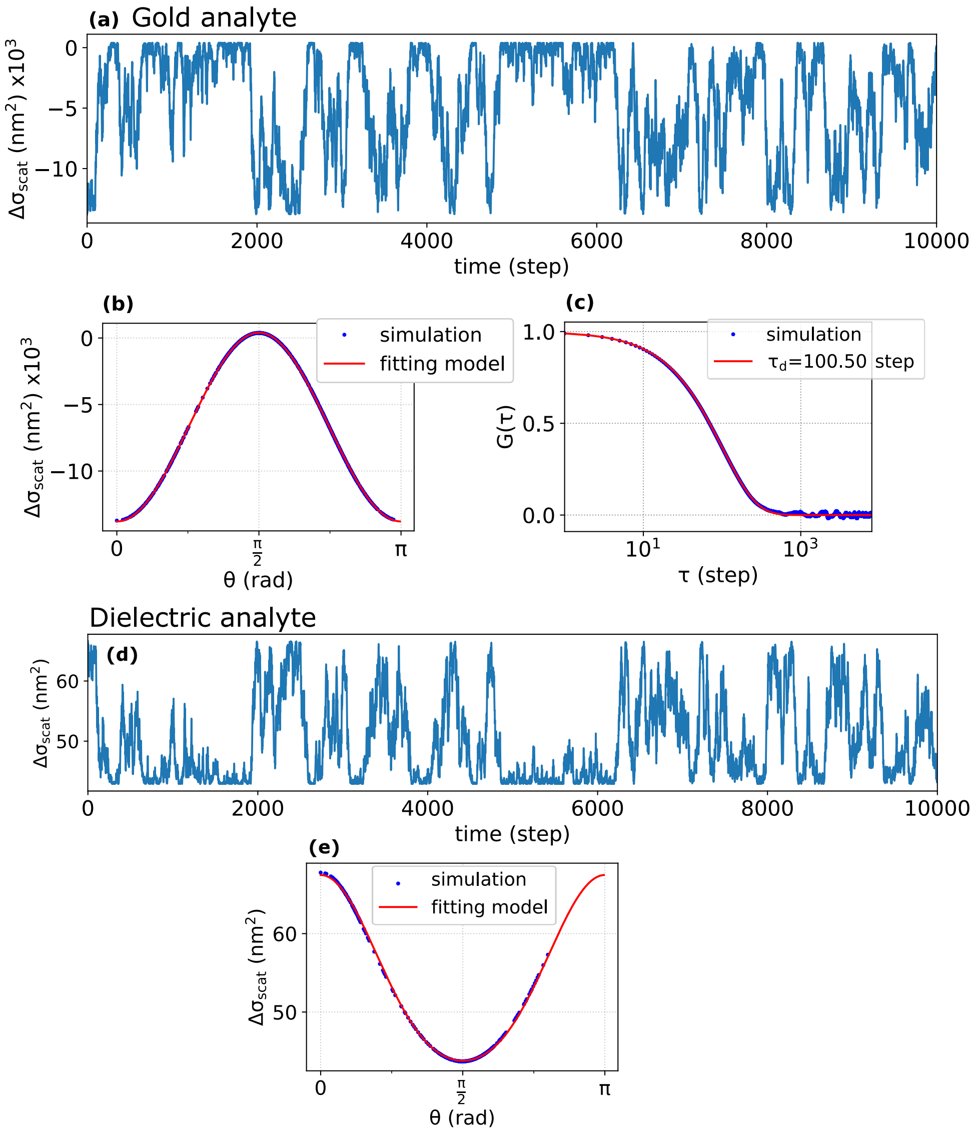

Figure S1: Orientational random walk simulation of a small nanorod ($22{\times}5\text{nm}^2$) near the tip of a sensor GNR ($112{\times}40\text{nm}^2$) with $x=16\,\text{nm}$ surface to center distance. **a)** Scattering cross section of the GNR-rotating-nanorod system as a function of time (step of the random walk). The negative changes of the scattering cross section $\Delta\sigma_{scat}$ are due to the splitting of the resonance peak (by considering the unperturbed sensor GNR as a reference). **b)** Dependence of the signal amplitude on the polar angle of the rotating nanorod θ, showing the ($Acos^4\theta + Bcos^2\theta$) dependence. **c)** Calculated autocorrelation of the trace shown in (a) for 10^6 steps. The red curve is the single-exponential fit with decay time τ_d=100.5 steps. Comparing the mean value of the decay time histogram of fig.4 in the main text, each step can be considered equivalent to ≈0.02 µs. **d)** Same calculations for a dielectric rotating nanorod, this time $\Delta\sigma_{scat}$ is 500 times smaller and purely positive. **e)** Dependence of the signal amplitude on the polar angle of the rotating nanorod θ, again showing a dependence of the form ($Acos^4\theta + Bcos^2\theta$). This time, however, the dominant part is the squared term $cos^2\theta$ rather than $cos^4\theta$. Indeed, for a dielectric rotating nanorod, the dipole coupling effect is much weaker than for the gold nanorod and non-resonant one.

S1.2 Mode splitting

As shown in fig.3c in the main text, plasmonic coupling of a resonant small nanorod to the sensor GNR creates a splitting in the spectrum. We have done a calculation of scattering cross section σ_{scat} for a small nanorod with $18{\times}5$ nm^2 dimensions (which is the dimension of the second sample of small nanorods according to the manufacturer's data sheet). The calculations have been done for incident linearly polarized light with polarization along the main axis of the sensor GNR and an incident wavelength of 770 nm.

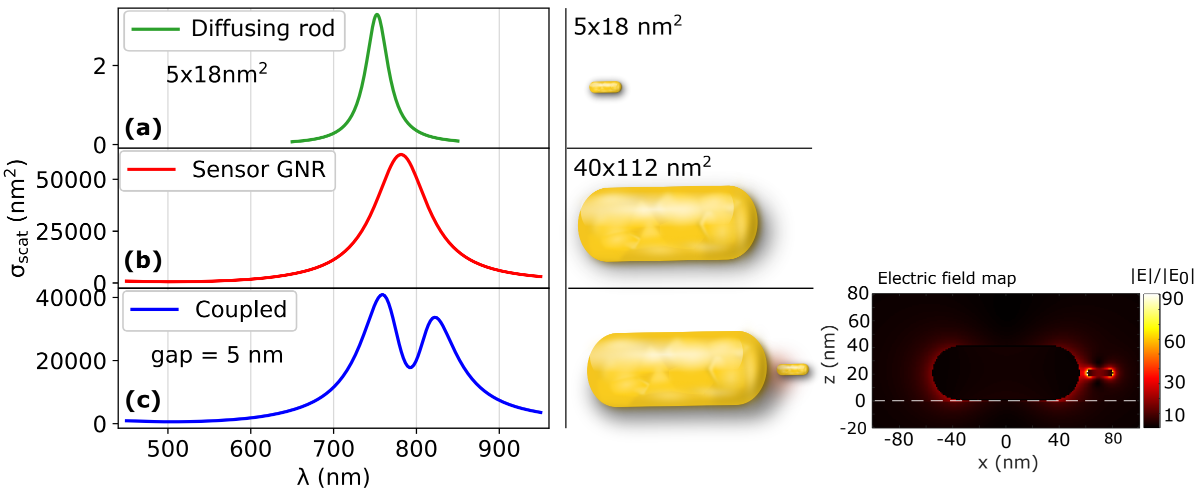

Figure S2: Calculated scattering cross section of **a)** A small nanorod (green curve) corresponding to the second sample (780 nm LSPR). **b)** $112{\times}40$ nm^2 GNR as the sensor (red curve). **c)** Coupled system (blue curve) with 14 nm surface-to-center distance. The spectrum shows a clear mode splitting due to the plasmonic mode coupling. Notice that, although the scattering cross section of the small nanorod (green) is extremely small in comparison to the sensor GNR (red), it has a considerable effect on the mode splitting (blue).

S1.3 Angle-dependent mode splitting

To investigate the effect of the rotating-rod angle with respect to the sensor GNR, we repeat the same calculation for different angles θ of the rotating rod. θ is the polar angle of the rotating rod with respect to the main axis of the sensor GNR. We then plot the deviation of the scattering cross section from the unperturbed sensor GNR's as $\Delta\sigma_{scat}$. The results shown in fig. S3 reveal the angle dependence of the coupling effect. At some angles ($\pi/2$

and $3\pi/2$) the deviation of the σ_{scat} from the unperturbed sensor GNR is negligible (white regions in fig. S3a). At these angles, there is no coupling effect.

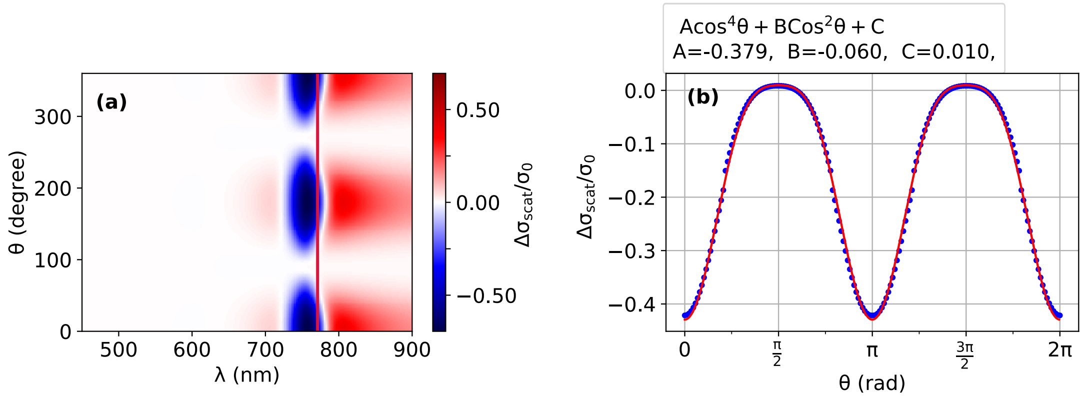

Figure S3: Simulated scattering cross section of a fixed sensor GNR ($112\times40\text{nm}^2$) and a ($18\times5\text{nm}^2$) rotating rod. The rotating rod is located near the tip and on the axis of the sensor GNR at 14 nm surface-to-center distance. **a)** Color graph where intensity corresponds to the normalized difference of the scattering cross section of the coupled system from the scattering cross section of the unperturbed sensor GNR ($\Delta\sigma_{scat}/\sigma_0$). The x axis is the wavelength and the y axis is the polar angle (θ) between the long axis of the rotating rod and the sensor GNR's axis. Rotating the nanorod around the fixed axis (long axis of the sensor GNR) with polar angle θ, we find clear intensity changes, which are maximal for specific wavelengths (in this case between 700 to 850 nm due to the mode splitting). The crimson vertical line shows 770 nm wavelength, the probe wavelength in our measurements. **b)** Intensity line along the crimson line in figure (a), corresponds to 770 nm wavelength. The blue dots show the variations of the scattering cross section of the coupled system with the polar angle θ. The fitted curve (red curve) is $Acos^4\theta + Bcos^2\theta + C$ which is compatible with the analytical model.

To compare the splitting effect for different rotating-rod samples used in this work (with LSPRs of 700, 780 and 850 nm), we repeated the same calculation as fig. S3a for different rotating rods. As fig. S4 shows, the splitting also depends on the size of the rotating rod. By increasing the length of the rod (from **a** to **c**) the mode (blue region) shifts to longer wavelengths. The vertical line in fig. S4 indicates the probe wavelength (770 nm) which shows positive deviation ($\Delta\sigma_{scat}$) for the shortest rod (a) with 700 nm LSPR. In real measurements, due to the size distribution of the rods and heterogeneity in the position relative to the sensor GNR, negative deviations are possible for the shortest rotating rod.

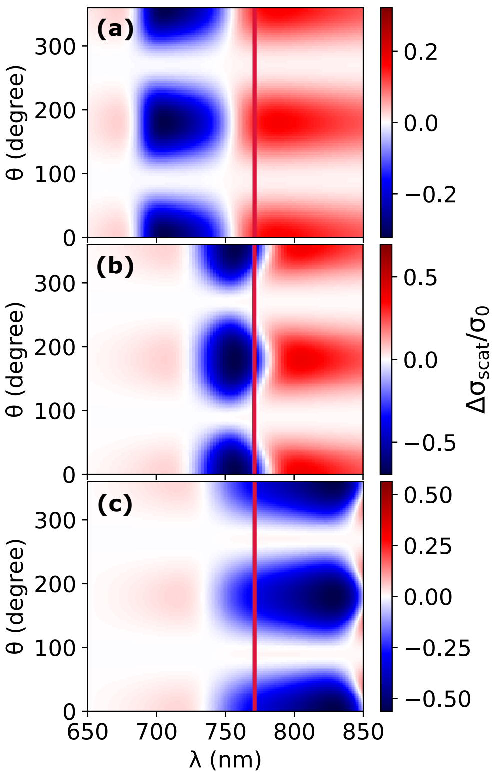

Figure S4: Simulated scattering cross section of a fixed sensor GNR ($112{\times}40\text{nm}^2$) and a rotating rod with dimensions of **a)** $15{\times}5\text{nm}^2$, **b)** $18{\times}5\text{nm}^2$ and **c)** $22{\times}5\text{nm}^2$. The splitted modes shift to longer wavelengths upon increasing the aspect ratio of the rotating rod. The vertical line shows the probe wavelength 770 nm. At this probe wavelength, the probability of having positive events is largest for the short rod $15{\times}5\text{nm}^2$.

S1.4 Randomly rotating rod

In fig. 2a in the main text, we investigate the effect of the distance x on the coupling for a fixed angle $\theta = 0$. Plot (c) of the same figure shows how the distance x affects the angle dependence of the scattering cross section σ_{scat}. The plots have been fitted with a polynomial of $cos^2\theta$. In this section, we investigate the coefficients of the polynomial as a function of distance.

Dependence of the polynomial coefficients in cos$^2\theta$ on the distance between the sensor GNR and the rotating rod:

Here, we investigate how the coupling strength changes with the distance of the rotating rod from the sensor GNR and consequently how it affects the angle dependence of the

scattering cross section. We repeated the simulation of fig. S1 for $22\times5\text{nm}^2$ rotating rod. For different distances, we calculate the fitting parameters of fig. S1b in the main text by $Acos^2\theta + Bcos^4\theta + Ccos^6\theta$ function. The result in fig. S5 shows that, for close distances, the contribution of higher powers is dominant, because the coupling is stronger. At large distances, the lower-order term $cos^2\theta$ dominates the angle dependence of the scattering. We also investigate extremely close distances (less than 5 nm surface to surface distance), at such close distances, higher orders in $cos^2\theta$ appear in the angle dependence of the scattering. By fitting with $Acos^2\theta + Bcos^4\theta + Ccos^6\theta$, we see how the $cos^6\theta$ contributions increases at short distances (fig. S5c).

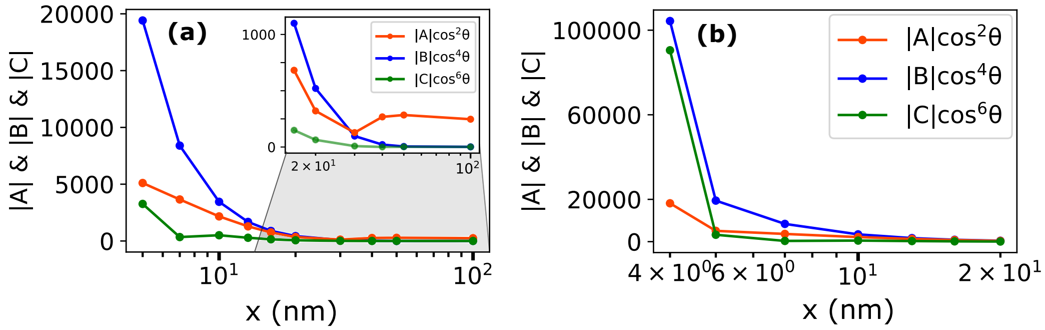

Figure S5: **a)** Fitted parameters of the simulated results shown in fig. 4c (main text) for different distances of the rotating rod and sensor GNR. The fitting function is $Acos^2\theta + Bcos^4\theta + Ccos^6\theta$. The dimensions of the rotating rod are $19.1\times5.9\text{nm}^2$. By decreasing the distance to less than 5 nm the absolute value of C strongly increases. **b)** To be more clear, we have plotted the small distances in this separate plot.

Rotational autocorrelation decay time from the random walk simulation

By simulating the time trace of the scattering cross section we calculate the autocorrelation curve and fit a single exponential function to it. We repeat the simulation for different distances, the results are shown in fig. 2d in the main text, for three different wavelengths: 720, 785 and 800 nm.

S1.4.1 Effect of the sensor-rotating-rod gap

We performed the random orientation simulation and calculated the scattering cross section for different gap distances (metal-to-metal distance of the sensor-rotating-rod). Figure. S6 shows the deviation of the scattering cross section for two different gaps. Gap =6 nm corresponds to negligible coupling, therefore, the angle-dependence scattering is similar to the confocal case (we have fitted with $cos^2\theta$). We also have compared it with the corresponding of confocal simulation in (c) which shows three orders of magnitude higher sensitivity for near-field simulation (d and e) in comparison to the confocal in simulation (c).

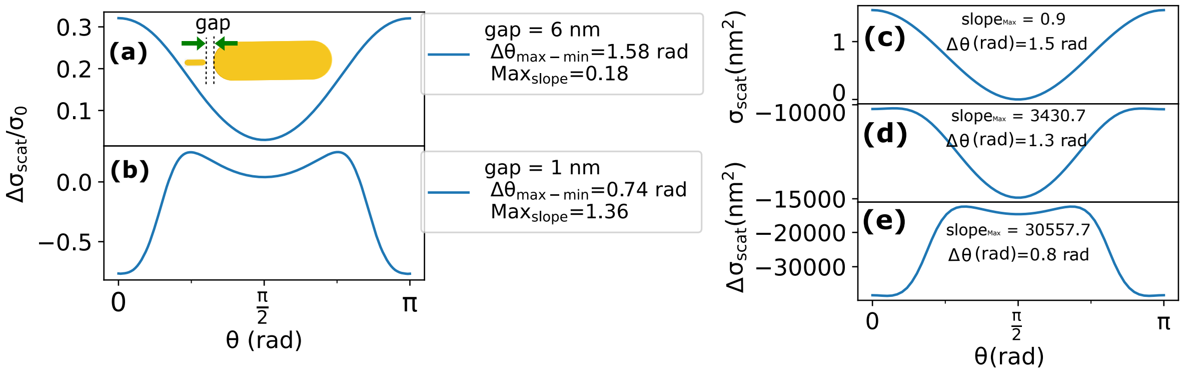

Figure S6: **a, b)** Angle dependence of the scattering cross section $\Delta\sigma_{scat}/\sigma_0$, calculated from a rotating rod $18{\times}5\text{nm}^2$ at a fixed point near the tip of a sensor GNR ($112{\times}40\text{nm}^2$) with **a)** 6 nm and **b)** 1 nm surface-to-surface distance. Note the much steeper angular dependence at short distances, for a specific angle. **c)** Angle-dependence scattering cross section of a rotating rod without any sensor GNR, excited by a linearly polarized plane wave corresponding to the confocal measurement. **d, e)** The same plots as (a) and (b) without normalization to compare with (c).

S1.5 Plasmonic dipole-dipole coupling (theory)

When two gold nanorods approach each other, their individual dipole modes couple and yield two eigenmodes at different energies. We can write the induced dipole moments of the sensor GNR ($\vec{p}_S$) and the rotating rod ($\vec{p}_A$) as:

$$\vec{p}_S = \overleftrightarrow{\alpha}_S(\vec{E}_0 + \overleftrightarrow{\phi}_{AS}\,\vec{p}_A) \quad and \quad \vec{p}_A = \overleftrightarrow{\alpha}_A(\vec{E}_0 + \overleftrightarrow{\phi}_{SA}\,\vec{p}_S), \tag{1}$$

where $\vec{E}_0$ is the incident electric field, $\overleftrightarrow{\alpha}_S$ and $\overleftrightarrow{\alpha}_A$ are the polarizability tensors of the sensor GNR and rotating rod, respectively. $\overleftrightarrow{\phi}_{AS}$ is the electric-field tensor created by the rotating rod at the sensor GNR position and $\overleftrightarrow{\phi}_{SA}$ is the electric-field tensor created by the sensor GNR at the rotating rod position. We consider the orientation of the incident electric field $\vec{E}_0$ and the long axis of the GNR along z. Then, we can solve the $\vec{p}_S + \vec{p}_A = \overleftrightarrow{\alpha}_{eff}\vec{E}_0$, where $\overleftrightarrow{\alpha}_{eff}$ is the effective polarizability of this coupled system. By using the Bra-ket notation, neglecting retardation, and considering $\overleftrightarrow{\alpha}_A = \alpha_A |\hat{u}\rangle \langle\hat{u}|$, $\overleftrightarrow{\alpha}_S = \alpha_S |\hat{z}\rangle \langle\hat{z}|$ and $\overleftrightarrow{\phi}_{AS} = \overleftrightarrow{\phi}_{SA} = \frac{1}{4\pi\epsilon_0 r^3}(3|\hat{r}\rangle\langle\hat{r}| - 1) = \phi_0(3|\hat{r}\rangle\langle\hat{r}| - 1)$, we can write the total dipole moment of the coupled system ($\vec{p} = \vec{p}_S + \vec{p}_A = \overleftrightarrow{\alpha}_{eff}\vec{E}_0$) as:

$$\overleftrightarrow{\alpha}_{eff} = \alpha_S + (1 + 2\alpha_S\phi_0 |\hat{z}\rangle\langle\hat{z}|)(1 - 4\alpha_A\alpha_S\ \phi_0^2\ cos\theta\, |\hat{u}\rangle\langle\hat{u}|)^{-1}\alpha_A(|\hat{u}\rangle\langle\hat{u}| + 2\alpha_S\phi_0 cos\theta\, |\hat{u}\rangle\langle\hat{z}|) \tag{2}$$

Then, by considering the linearly polarized incident light along z and using a Taylor expansion for $(1 - 4\alpha_A\alpha_S\ \phi_0^2\ cos\theta\, |\hat{u}\rangle\langle\hat{u}|)^{-1}$ the total dipole moment can be written as:

$$\vec{p} \approx \alpha_S E_0 |\hat{z}\rangle + \alpha_A E_0(1 + 2\alpha_S\phi_0)(1 + 4\alpha_A\alpha_S\ \phi_0^2\ cos^2\theta)[2\alpha_S\ \phi_0\ cos^2\theta\, |\hat{z}\rangle + cos\theta\, |\hat{u}\rangle] \tag{3}$$

For strong coupling in the near field, the product $(\alpha_S\phi_0)$ of sensor polarizability and field tensor is considerably higher than 1, so that the $cos^2\theta$ term dominates and we can neglect the $cos\theta$ term. Therefore, the dipole moment of the coupled system writes approximately as a power series of $cos^2\theta$.

S1.6 Side-by-side interaction

Plasmonic coupling also occurs in a side-by-side configuration of the sensor-rotating-rod system (schematics in fig. S7a and c). Figure. S7a shows a rotating rod in a fixed position

near the long axis of the sensor GNR (with 5 nm gap). Figure S7c shows variation of the position of the rotating rod along the long axis of the sensor GNR, while the angle $\theta = 0$ is fixed. We have calculated the deviations of the scattering cross section of the sensor-rotating-rod system (with respect to the unperturbed GNR) $\frac{\Delta\sigma_{scat}}{\sigma_0}$ as a color-coded graph in figs. S7b and d. The dimension of the sensor GNR is $112\times40\text{nm}^2$ and that of the rotating rod is $18\times5\text{nm}^2$. The graph in (b) shows the calculated $\Delta\sigma_{scat}/\sigma_0$ for a wavelength range from 650 to 850 nm. Sweeping the angle θ yields variation of the scattering cross section from almost zero deviation for $\theta = 90°$ and maximum deviation for $\theta = 0$ (for a wavelength range from 650 to around 760 nm). In the color graph of (d) $\Delta\sigma_{scat}/\sigma_0$ is shown for a fixed angle $\theta = 0$ and sweeping position from $z = -20$ nm to 20 nm (metal-to-metal gap is 5 nm). Therefore, the sub-events in the experimental scattering time trace (same as fig. 3d in the main text) can be due to the freely diffusing single analytes through the near field.

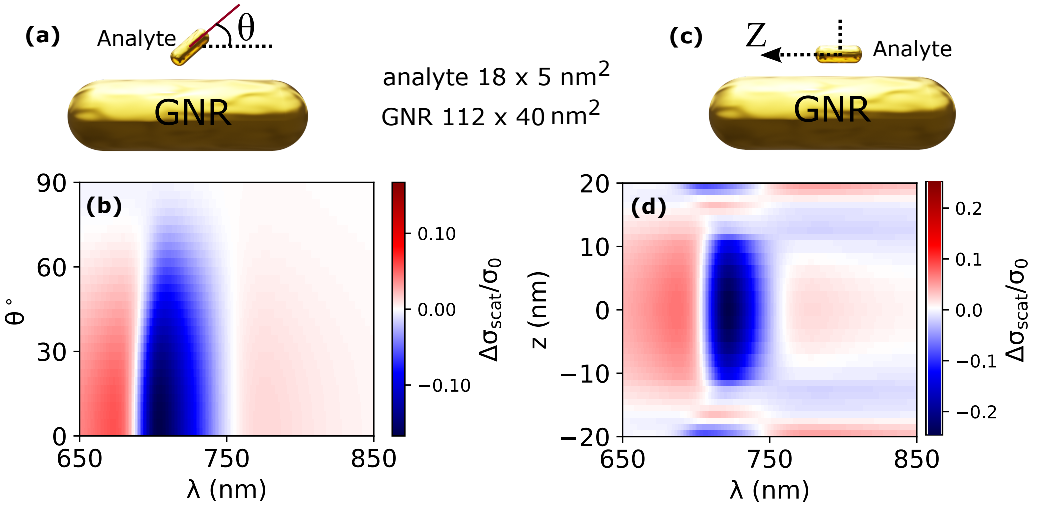

Figure S7: **a)** Schematic of the sensor-rotating-rod system with θ as polar angle of the rotating rod with respect to the long axis of the GNR. Surface-to-center distance is 14 nm and the origin is located at the middle of the sensor GNR. **b)** Calculated deviation of the scattering cross section of the sensor-rotating-rod system with respect to the unperturbed GNR by sweeping the θ angle. **c)** Schematic of the sensor-rotating-rod system. The gap (surface-to-surface distance) is 5 nm. **d)** Calculated deviation of the scattering cross section of the sensor-rotating-rod system with respect to the unperturbed GNR by sweeping the rotating rod's position along the long axis of the sensor GNR (Z).

S2 Optical setup

In this work, we have used a confocal microscope (fig. S8) to measure the scattering time trace of an immobilized sensor gold nanorod (GNR) on a glass substrate. We probe the sensor rod with linearly polarized (770 nm wavelength) light reflected from a beam-splitter with a small angle (7°) toward a high NA (1.3) oil immersion objective. The reflected and scattered light are collected through the same path and detected by means of a high-speed avalanche photodetector (APD, max. bandwidth 400 MHz) with 50 MHz sampling rate. The linear polarization of the incident and collected light is tunable by a rotatable $\lambda/2$-plate and a Glan-Thompson polarizer to excite the longitudinal localized plasmon resonance of the sensor rod. The spectrum of the sensor rod can be recorded by exciting with a white-light source and dispersing the scattered light collected with the same objective in a spectrograph.

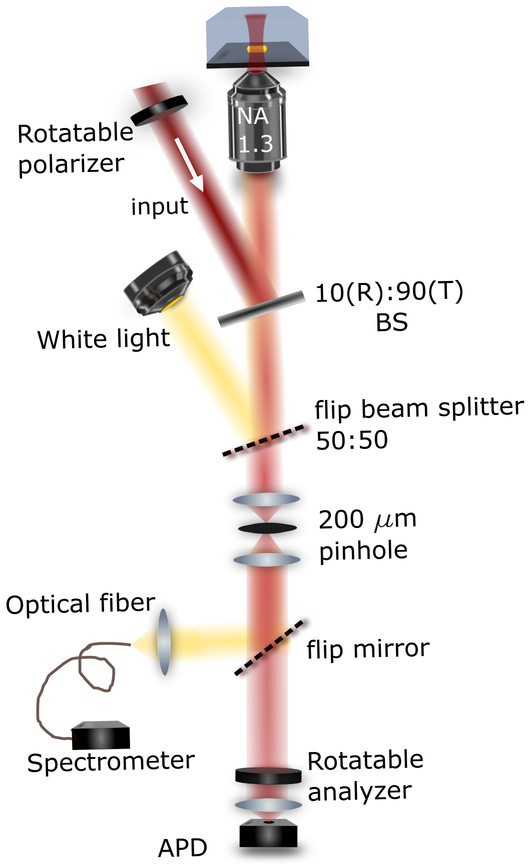

Figure S8: Confocal microscope that has been used in this work. Linearly polarized light is reflected from a 10:90 beam splitter and reaches an oil immersion objective, which collects the reflected and scattered light. The collected light is transmitted through the beam splitter and a 200 μm pinhole, sent to an APD through a polarizing analyzer. The scattering spectrum of the sensor GNR is excited by a broad-band white light source which enters the optical path of the microscope through a flip 50:50 beam splitter. The collected light is sent into the spectrometer through a flip mirror and an optical fiber.

S3 Diffusing rods

The diffusing rods used in this work have been purchased from Nanopartz as: A12-5-700-CIT-DIH-1-25, A12-5-780-CIT-DIH-20-1 and A12-5-850-CIT-DIH-1-25.

S3.1 SEM

We have done scanning electron microscopy (SEM) measurements of these three groups of rods. To prepare the samples for SEM, we clean the ITO coated glasses by sonicating in acetone for 40 min and ethanol for 30 min and rinse with milli-Q water. Then, we UV-clean them for 30 min. We sonicate the gold nanorod stock solutions for 5 min and rinse the substrates with ethanol/hydrochloric acid (HCl 100 mM) with 90/10 volume ratios. We mix 90 µL of gold nanorod stock solution with 10 µL 100 mM HCl to bring the pH to 2 and incubate it on the ITO coated glasses for 60 min. As the last step, we rinse the samples with ethanol/HCl (100 mM) and incubate for 15 min to make sure the analytes have been immobilized, then finally rinse with milli-Q water and dry with nitrogen. We have done SEM measurements with 15 kV and 50 pA, the results are shown in fig. S9.

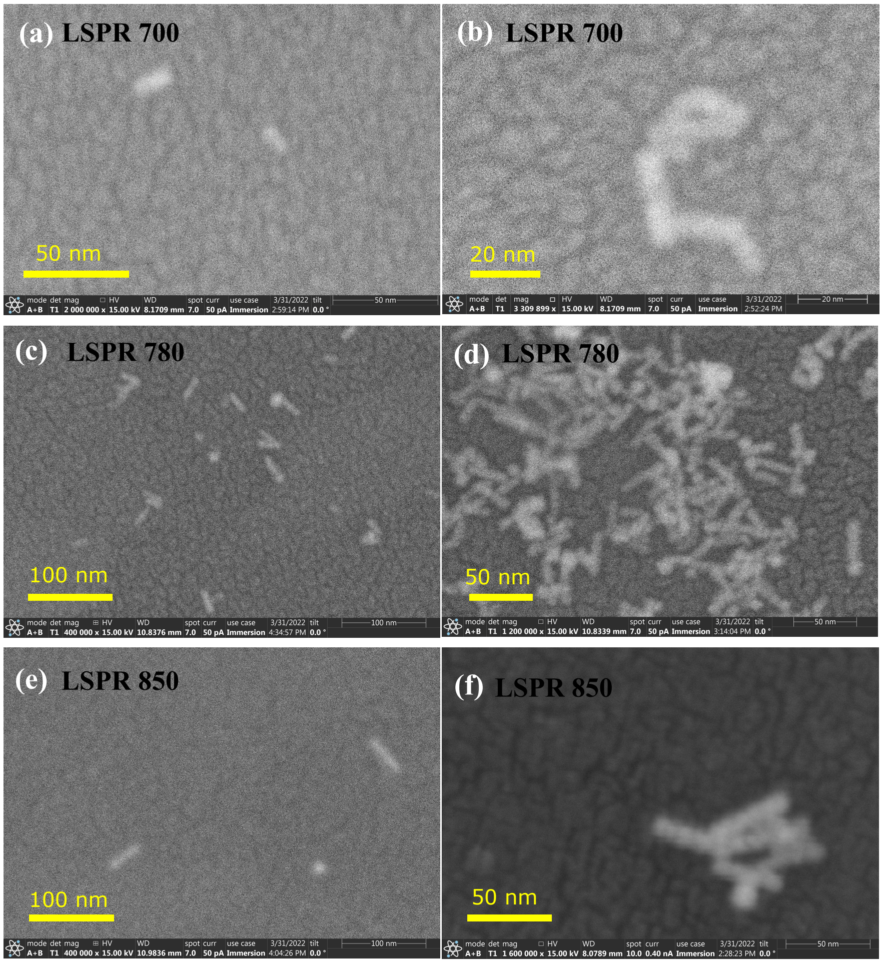

Figure S9: SEM images of the (small) gold nanorods with three different Localized Surface Plasmon Resonance (LSPRs), consequently different aspect ratios. **a, b)** LSPR = 700 nm, **c, d)** LSPR = 780 nm, **e, f)** LSPR = 850 nm.

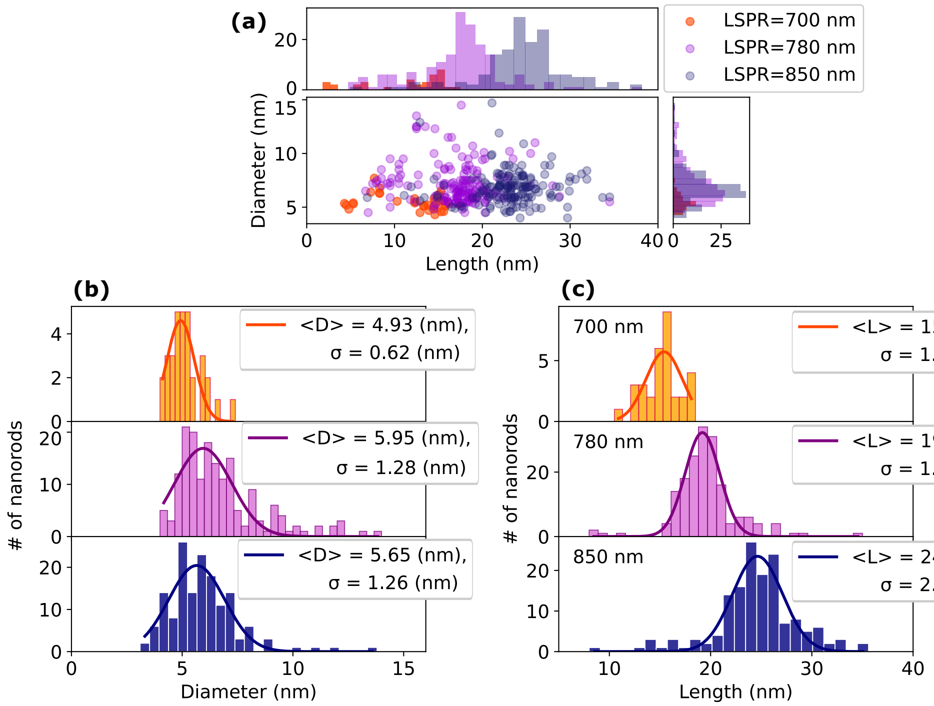

Figure S10: Size distribution of three groups of rods extracted manually from SEM images (Orange: LSPR=700 nm, violet: LSPR=780 nm, dark blue: LSPR=850 nm). **a)** Scatter plots of diameter vs length of the nanorods shows the overlap of the size distributions of these three group of nanorods. Notice that some of the particles are spheres (Length=Diameter). We have ignored them to calculate the average diameter and length in the following histograms. **b)** Histograms of diameter distributions fitted with Gaussians with mean values of 6.7, 7.8 and 7.1 nm, respectively for sample 1, 2 and 3. **c)** Length distribution for three groups of samples with fitted Gaussian curves, showing 15.4, 19.1 and 24.6 nm as mean values. The numbers of nanorods are 36 nanorods for the first solution (LSPR=700), 173 nanorods for the second one (LSPR=780) and 152 nanorods of the third one (LSPR=850 nm).

S3.2 Spectrum

We also measured the ensemble spectra of the diffusing-nanorod samples. The results are shown in fig. S11 for three samples of nanorods.

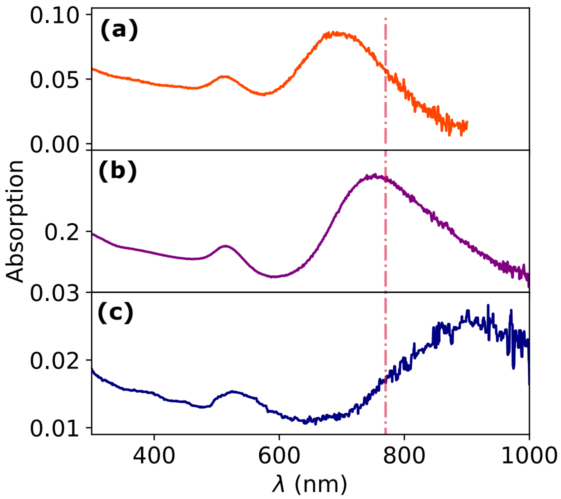

Figure S11: Spectra of stock solutions of the diffusing nanorods as an ensemble measurement which show clear peaks of longitudinal and transverse LSPR. The dash-dotted line shows the probe wavelength (770 nm) in our measurement. **a)** A12-5-700-CIT-DIH stock solution. **b)** Stock solution of A12-5-780-CIT-DIH with clear longitudinal LSPR peak at around 760 nm. **c)** A12-5-850-CIT-DIH stock solution, which shows 880 nm as the resonance peak. The transverse LSPR for all the samples is around 520 nm.

S4 Temperature estimation around the sensor GNR

Here, we rule out the possibility for heating-induced reshaping of our sensor GNRs. In our measurement the power in the back-focal plane was 38 µW. To check for any possible thermal reshaping of the sensor GNR or sticking of the diffusing rods to it, we have recorded the spectrum of the sensor GNR before and after the measurement, and found no measurable change.

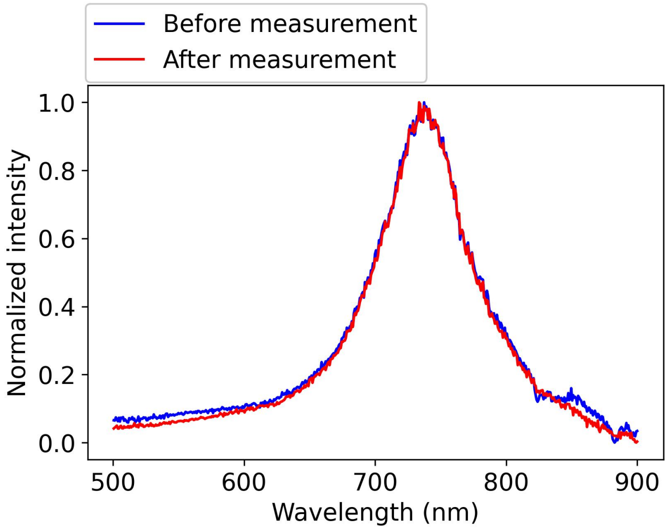

Figure S12: Measured spectrum of the sensor GNR before and after measurement (blue and red), indicating the absence of reshaping (blue shift) and of permanent sticking of the diffusing rods to the sensor GNR (red shift).

S4.1 Temperature simulation

In this subsection we discuss the temperature profile around the sensor GNR to investigate the viscosity response of the surrounding media (water). To calculate the temperature increase around the sensor GNR, we performed a COMSOL simulation (Fig. S13). We set the initial temperature to 20°C as that of our laboratory. According to the simulation there is 1 K temperature increase around the sensor GNR which cannot lead to any reshaping for a $112{\times}40\mathrm{nm}^2$ GNR.

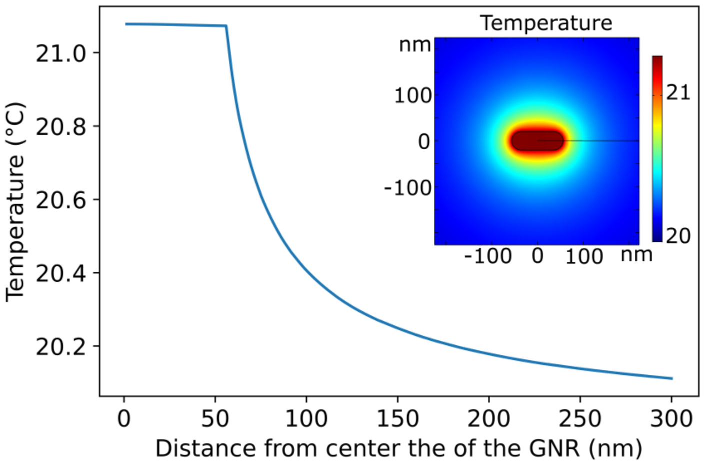

Figure S13: Simulated temperature field around the sensor GNR ($112 \times 40 \mathrm{nm}^2$) caused by an incident plane wave, linearly polarized along the long axis of the GNR with 770 nm wavelength and 38 µW power.

The refractive index changes of water due to 2.5 mM NaCl is less than 0.0001 (according to Larin *et al.*[3]). Therefore, we consider the surrounding environment as pure water. The refractive index changes of water by increasing the temperature by 1 K (from 20 to 21°C) is less than 0.01,[3] causing a viscosity change of 0.02 mPa s[4] around the GNR. This amount is too small to affect the rotational diffusion of the diffusing rods. Furthermore, we expect the thermophoretic effect in our measurement to be negligible.

S5 Time Resolution

To show an example of the smallest detectable fluctuation in our measurement (as a proof of high temporal resolution), we zoom in to one of the traces (in the main text), related to the 19.1 nm length diffusing rod.

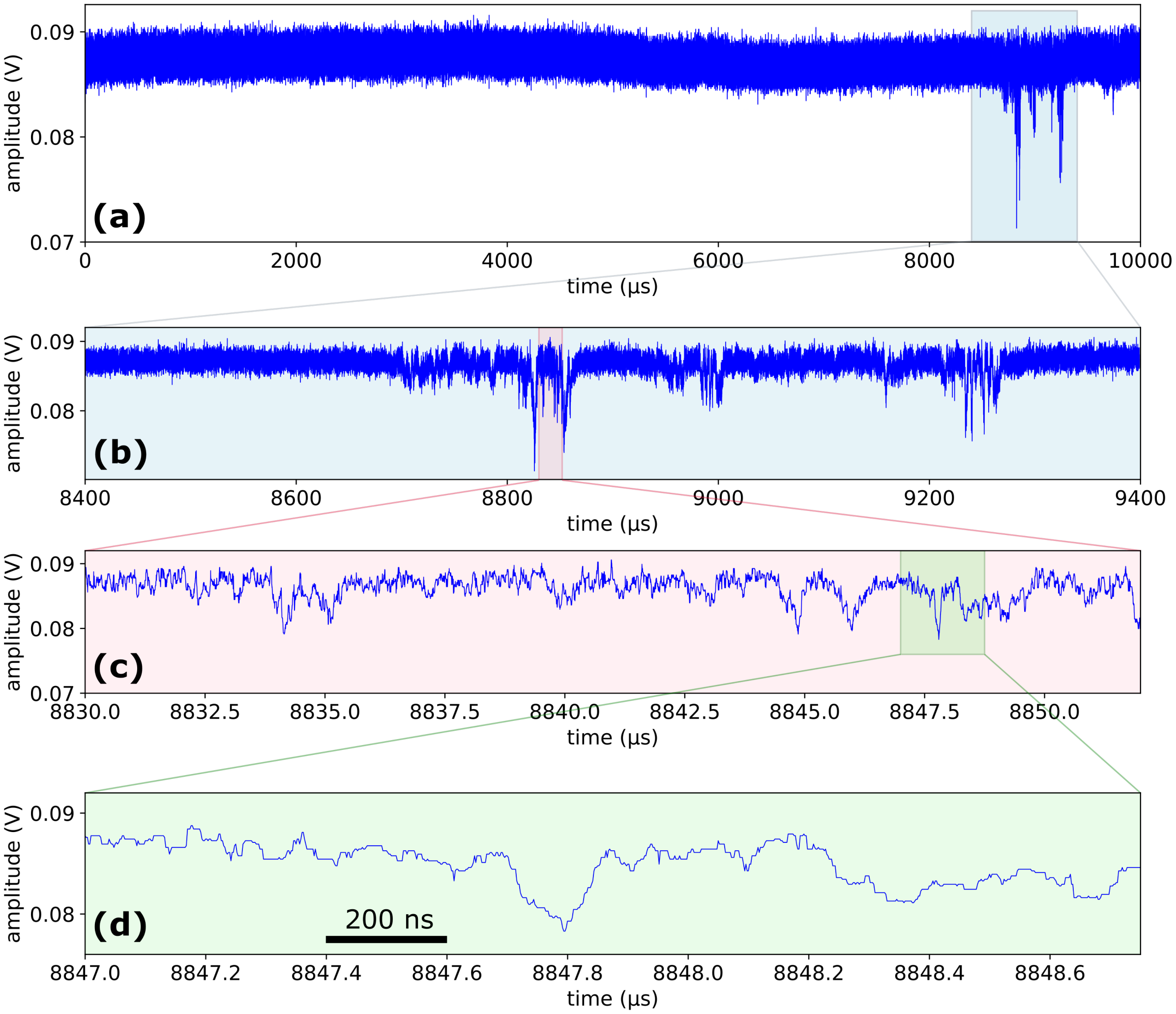

Figure S14: Measured scattering time trace of the sensor GNR 112×40nm^2 and an event between 8.4 to 9.4 ms related to a 19.1 nm length diffusing rod. By looking at the short sub-burst which occurs at 8847.8 µs, we see clear fall and rise of the signal with enough sampling points. The time resolution in our measurement, enables our method to detect such fast sub-bursts.

S6 More events with different diffusing rods

Here, we provide more examples of events for different diffusing rods.

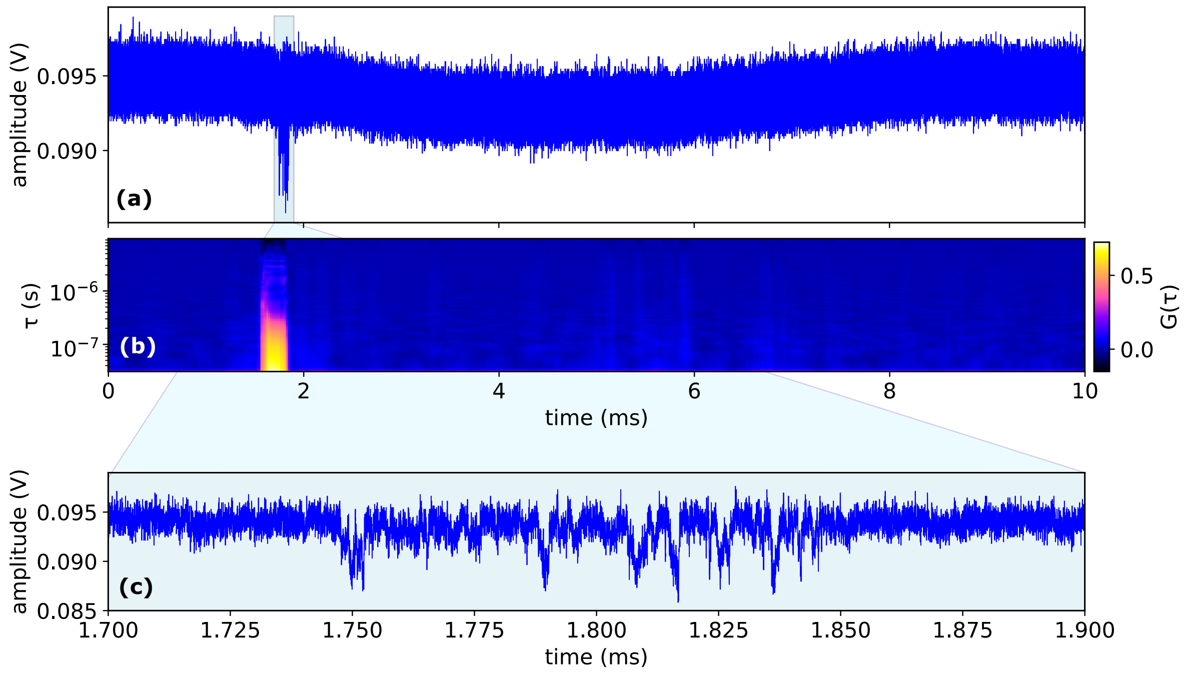

Figure S15: Single diffusing (15.5 nm length) rod detection in 2.5 mM NaCl. **a)** Scattering time trace of the sensor GNR. There is a clear event with negative sign between 1.7 to 1.9 ms which has been created by tumbling of a diffusing rod through the near-field zone of the sensor rod. **b)** Color map of the autocorrelation to specify the event, showing a clear contrast during the event. **c)** Zoom-in on the main part of the event with clear sub-bursts due to rotational diffusion of the nanorod.

We also have recorded some positive-sign events for this rod solution (15.5 nm length) in the same conditions. As the simulation shows in fig. S4 the sign of the event depends on the probe wavelength and the aspect ratio of the diffusing rod. Positive changes are more likely to occur for the first nanorod sample and 770 nm probe wavelength. Of course due to the size distribution and heterogeneity of interaction spots around the sensor GNR, there are also negative events for this sample as shown in S15. In the following figure we provide an example of a positive event for the first sample of diffusing rods. Figure S16d is the autocorrelation of the event shown in (c), which shows a large contrast in comparison to the autocorrelation of the background noise (in gray).

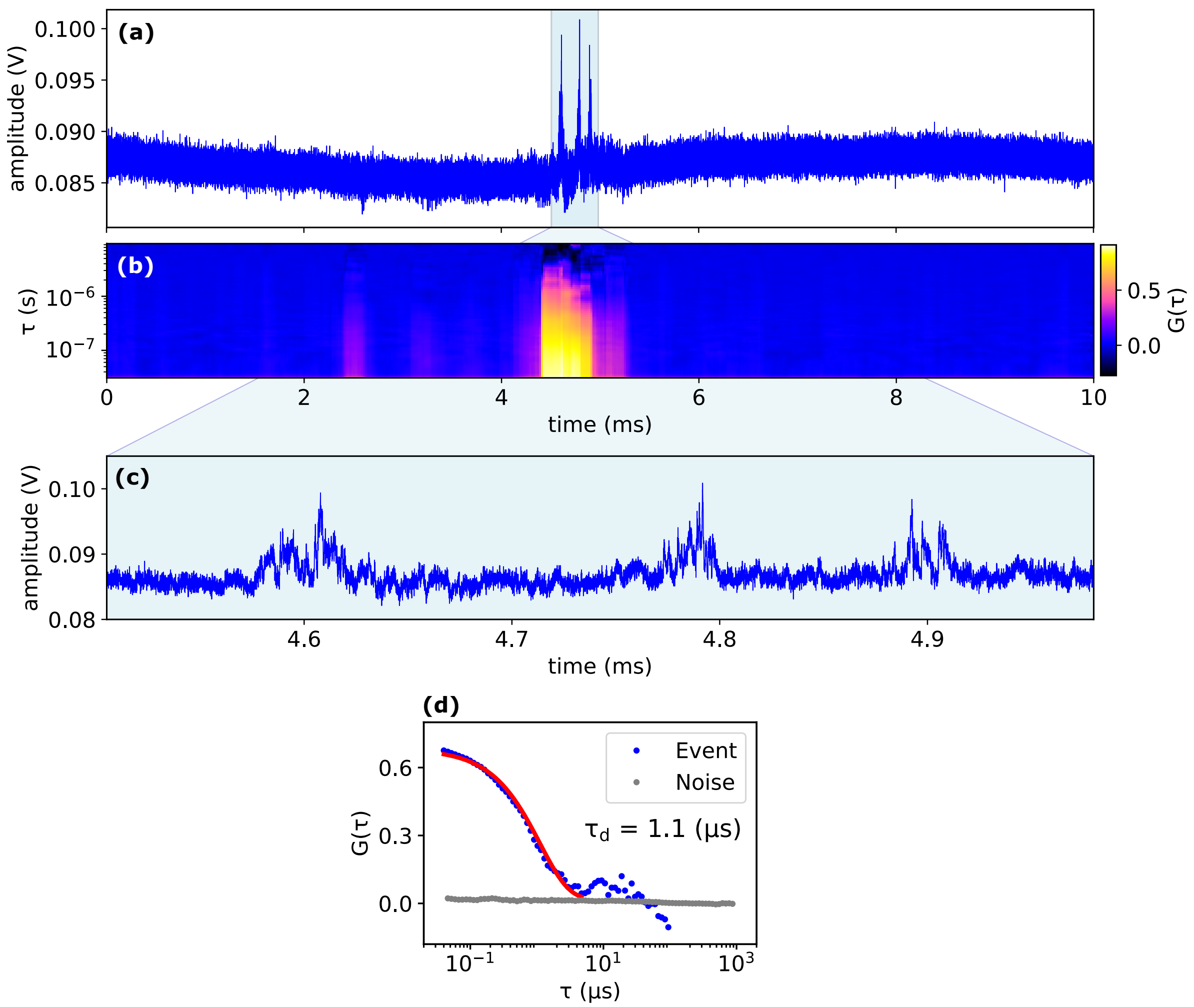

Figure S16: Scattering time trace of the sensor GNR under diffusion of 15.5 nm length rod sample. **a)** Positive event related to diffusion of a rod of the first sample through the near-field volume of the sensor GNR (see between 4.5 to 5 ms). The event shows three clear spikes which are probably due to the trajectory of the same diffusing rod through the near field multiple times. **b)** Color map of the autocorrelation showing clear contrast during the event. **c)** Zoom-in on the time trace of the event shows clear sub-bursts within each spike caused by rotational diffusion of the diffusing rod during its positional random walk. **d)** Autocorrelation of the event shown in (c) (blue dots). The red curve is the corresponding single-exponential fit with 1.1 µs as decay time. The gray dots are the autocorrelation of the background noise.

On average, the diffusion of nanorods from the third sample (24.6 nm length diffusing rod) through the near field yields comparatively higher amplitude events in the scattering time trace. Fig. S17 is an example of such an event. Due to the larger volume of these diffuisng rods, the coupling with the probe GNR is stronger which yields bursts with a

higher amplitude at the probe wavelength (770 nm) in comparison to other diffusing rods (for example in comparison to first rod sample in fig.S15).

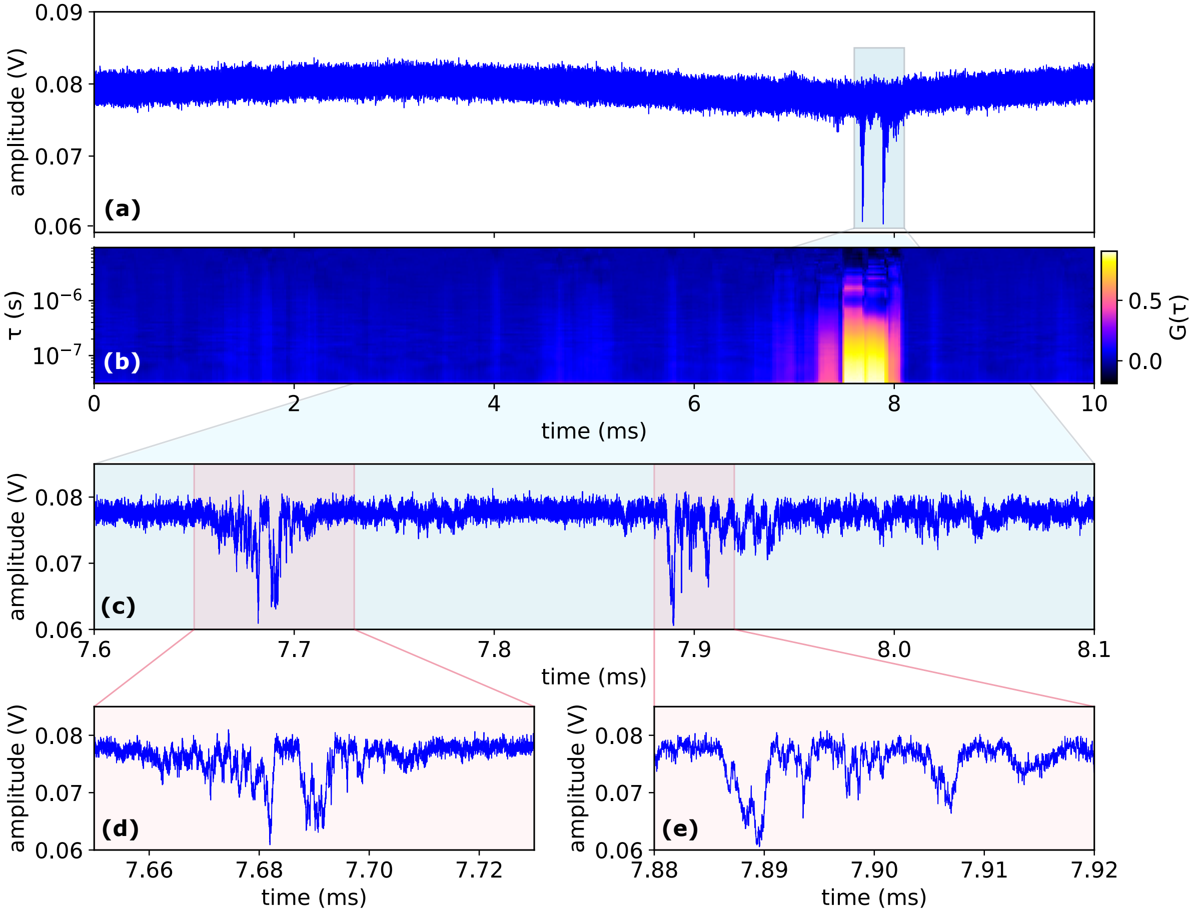

Figure S17: **a)** Scattering time trace of the sensor GNR which shows a clear event caused by a diffusing rod (with 24.6 nm length) through the near-field volume. The event shows two clear spikes which can be created by multiple diffusion of the same nanorod into and out of the near-field volume. **b)** Color map showing the autocorrelation trace for the time trace displayed in (a). **c-e)** Zoom-in on the event with clear sub-bursts during each burst caused by rotational diffusion of the nanorod.

In fig. 4(h-j) in the main text, each point corresponds to an event. High-amplitude A events caused by a stronger interaction of diffusing rod with the sensor have shorter rotational decay time τ_d. We showed examples of high amplitude events in fig. S14 to fig. S17. The autocorrelation is averaged over all sub-events. Weaker events as shown in fig. S18 contain a smaller number of sub-events, which can be due to the weaker diffusing-

rod-sensor interaction.

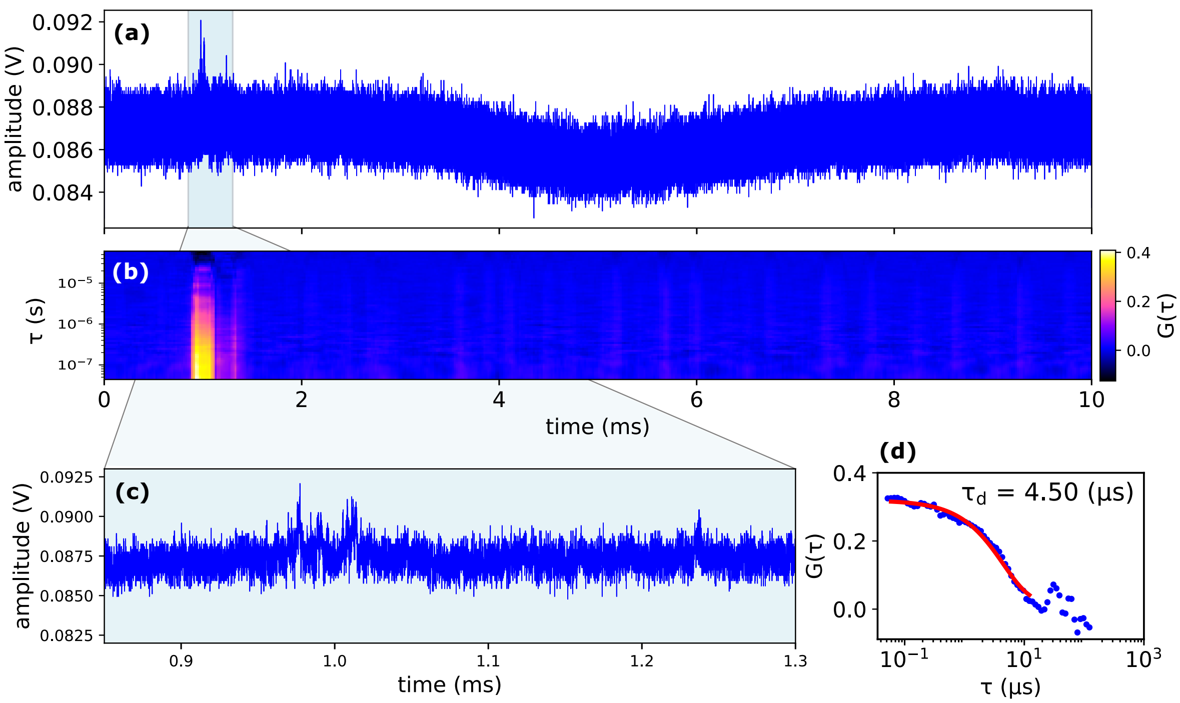

Figure S18: An example of weak events produced by the second sample of diffusing rods (19.1 nm length) **a)**. 10 ms scattering time trace of the sensor GNR which shows a weak event around 1 ms. **b)** Autocorrelation color map with clear contrast during the event. **c)** Zoom-in on the event with low-amplitude sub bursts. **d)** Autocorrelation of the highlighted event (blue dots) and the corresponding single-exponential fit (red) to the fast component with decay time τ_d =4.50 μs.

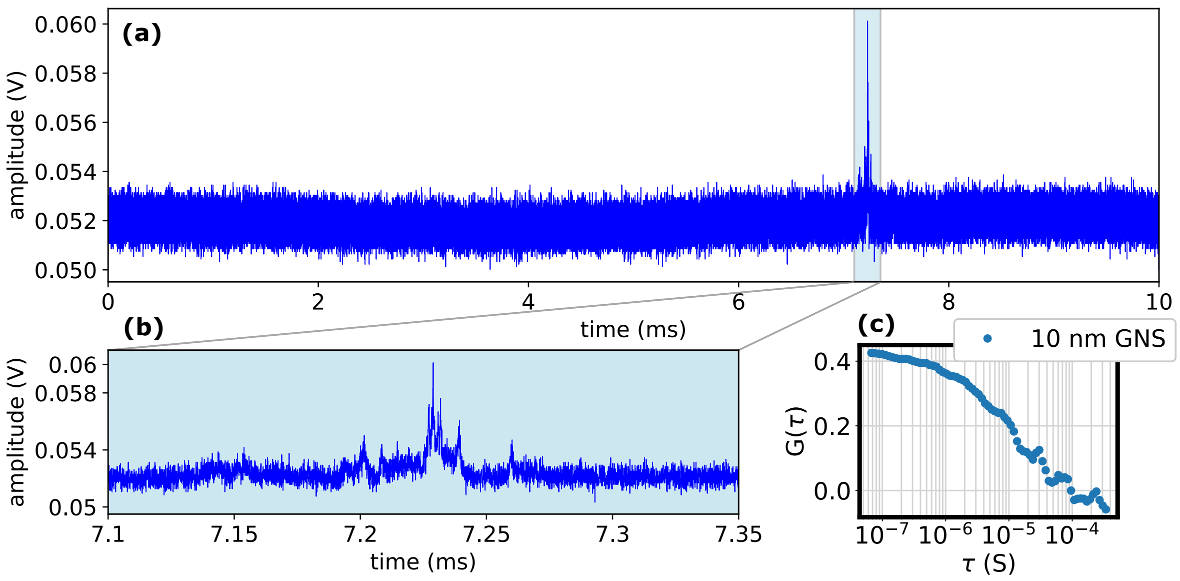

Figure S19: **a,b)** Event produced by a diffusing 10 nm gold nanosphere (GNS) around a sensor GNR, showing positive spikes and, in contrast to the diffusing nanorods, doesn't display a set of fast sub-bursts. Instead, a few comparably slow sub-bursts are created by diffusion of the same nanoparticle into and out of the near field. **c)** Autocorrelation of the trace shown in (a), which shows a single decay. Contrary to the anisotropic gold nanorods, there is no clear fast component due to the symmetry of the GNS.

S7 Translational diffusion

To estimate decay times due to translational diffusion, we have fitted a single exponential function to the slow component of the autocorrelation. The histograms of decay times τ_D have been plotted in fig. S20. The translational diffusion constant D_T of a rod is given by Tirado's theory, and by considering the additional friction due to the interface (glass substrate) by means of a damping coefficient F_z.[5] We can write:

$$D_T = \frac{k_B T}{3\pi\eta L F_z}(ln(p) + 0.312 + 0.565/p - 0.1/p^2) \quad (4)$$

where k_B is the Boltzmann constant, p is the aspect ratio (length divided by diameter L/d), T is the temperature and η is the solvent viscosity. We approximate the damping coefficient by considering $a \approx 5$ nm as the radius of an equivalent sphere with the same volume as our

big diffusing rod. We also have considered δ as the surface-to-surface distance of the diffusing rod with the glass substrate (by considering tip-to-tip configuration, $\delta \approx$ radius of the sensor rod - $a = 15$ nm). The damping coefficient F_z is:[5]

$$F_z = exp(0.0057685[ln(\delta/a)]^3 + 0.092235[ln(\delta/a)]^2 - 0.52669[ln(\delta/a)] + 0.76952) \approx 1.4, \quad (5)$$

therefore, the correction of the translational diffusion coefficient D_T for the biggest nanorod sample (diffusing rods) in our work is around 20%. This value is meant as an estimate for the effect's order of magnitude and cannot replace a more rigorous treatment of the proximity effect of the wall on the translational diffusion. Additionally, in general, the wall-effect damping on the rotation is less than the the wall effect on the translational diffusion.[6] So, we neglect the wall effect on the rotational diffusion.

We have extracted the translational diffusion time of our diffusing rods from experimental data by considering the decay time of the autocorrelation's slow component. The histograms of decay times have been shown in the following figure:

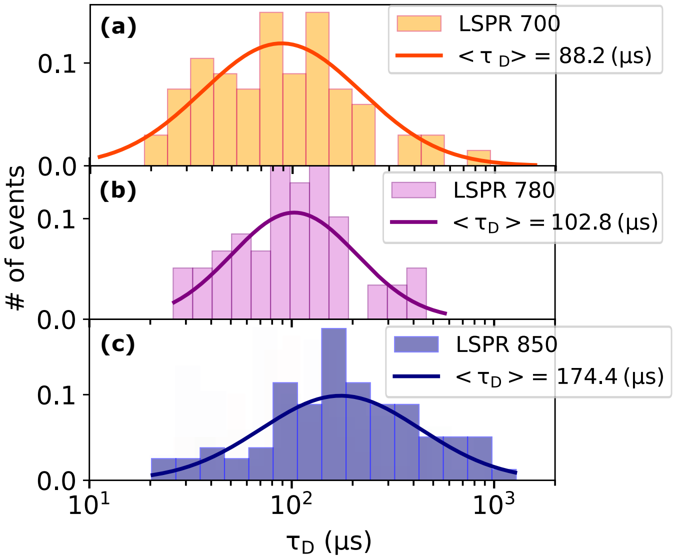

Figure S20: Decay time τ_D of the slow component of the autocorrelation, assigned to translational diffusion, for the three diffusing-rod samples, **a)** $15.4\times5\text{nm}^2$, **b)** $19.1\times5.9\text{nm}^2$ and **c)** $24.6\times5.6\text{nm}^2$.

The translational diffusion time is related by $\tau = r^2/(4D_T)$ to the diffusion coefficient and to r, the radius of the detection volume. For three samples of diffusing rods, the diffusion coefficients by considering 1 nm citrate layer are 2.84×10^{-11}, 2.38×10^{-11} and $2.13\times10^{-11} m^2/s$, respectively for 15.5, 19.1 and 24.6 nm length diffusing rods. By using the mean values of the fitted curves of the histograms as τ, we can find a detection length of around 90 nm, which is much larger than the expected extent of the near field. A possible explanation for this mismatch is the existence of an attractive potential between the sensor and the diffusing rods, which extends the dwell time of the diffusing rods in the vicinity of the sensor. According to Baaske *et al.*, the ionic strength of the solution is essential to shield repulsive interactions between particles and to detect events. We did not detect any events without electrolytes in the solution. However, in our work, an attractive force between different ligands (CTAB and citrate on the sensor and analyte rods, respectively) may cause some attractive interaction between the sensor and the diffusing nanorods. We speculate that the combination of repulsive and attractive forces extends the dwell time of the diffusing nanorods in the vicinity of the sensor, thereby leading to longer events and to an overestimation of the detection length (90 nm).

S8 Confocal monitoring of the rotational diffusion of diffusing rods

We compared our rotational diffusion measurements in the near-field to measurements in confocal microscopy, whereby we focused the laser at, or in the vicinity of, the glass substrate. The trace in fig. S21 is recorded at the glass interface with the second sample of diffusing rods (19.1 nm length diffusing rod). We find longer events and lower amplitude in comparison to the near-field events. The measurements have been done with the same probe wavelength as near-field measurements (770 nm), but with somewhat lower concentrations (resp. 7.0, 17, 8.3 nM) because of the larger confocal volume. We performed the confocal measurement

in the same conditions as the near-field one by collecting the reflected and scattered light in the same polarization orientation as the incident beam and focusing the laser on the glass substrate. The autocorrelation functions of the scattered intensity show a fast and a slower component in the range of a millisecond, which we assign to the translational diffusion through the confocal volume.

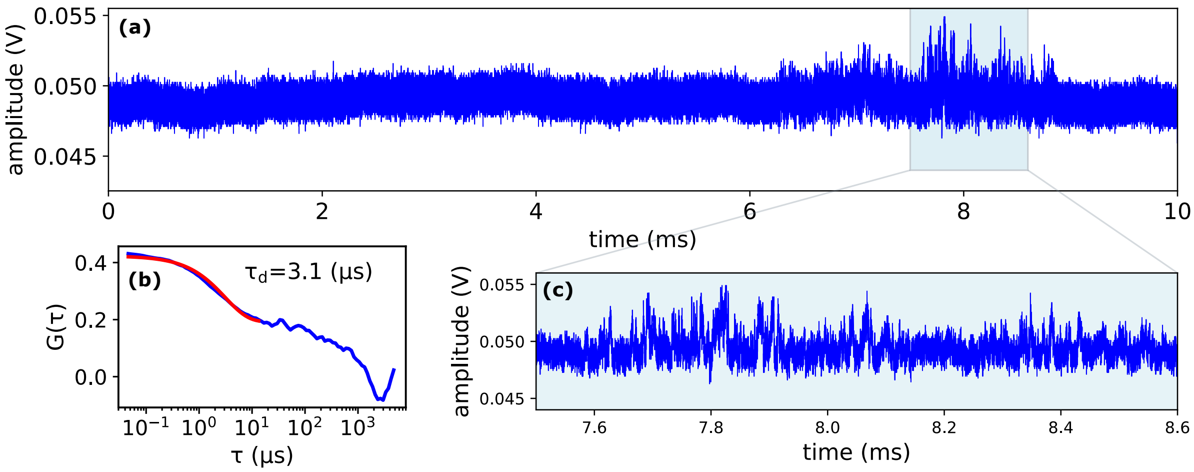

Figure S21: Example of a confocal measurement. **a)** Scattering time trace upon focusing the laser on the glass substrate with the second sample of diffusing rods (19.1 nm length). A comparatively long time event appears between 6 and 9 ms. **b)** Autocorrelation of the whole trace (blue) and single-exponential fit (red) with 3.1 µs decay time. **c)** Zoom-in on the event showing fast fluctuations within the event.

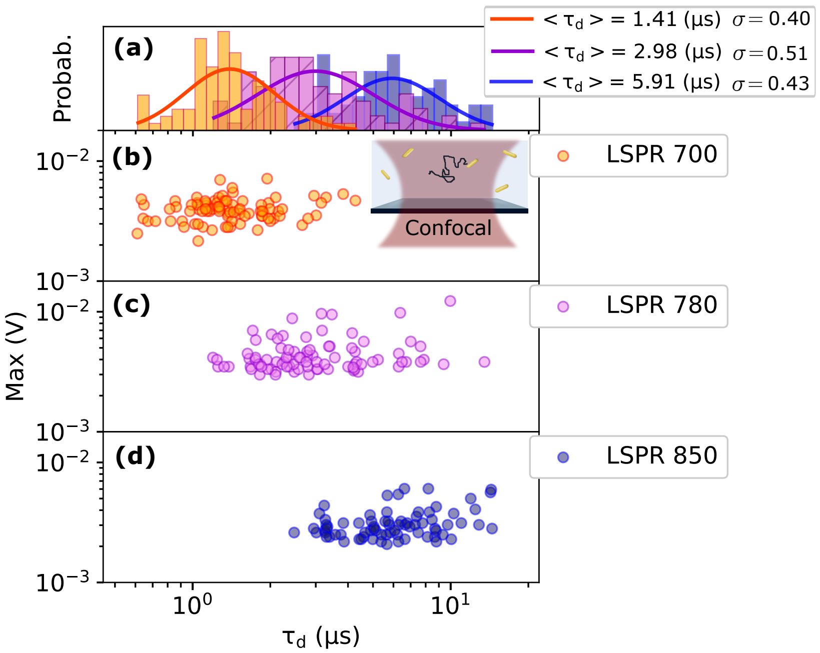

Figure S22: Scatter plots of maximum amplitude of each event versus rotational decay time (τ_d) in the confocal measurement for **a)** 15.5 nm, **b)** 19.1 nm and **c)** 24.6 nm length diffusing rods. The focus of the laser was located on the glass substrate. In contrast to near-field measurements (fig. 3h-j in the main text), we see no clear correlation between the intensity of the events and their rotational decay time.

S9 Angle sensitivity

To estimate the angle sensitivity, we consider the fastest fluctuations in each measurement associated to the largest amplitude in each event (as shown in fig. S23). We assign these fluctuations to orientation changes of the diffusing rod from 90° to 0° (with respect to the GNR's long axis), respectively providing no coupling and maximum coupling. This estimation is true if we assume that these fast changes are only due to angle changes. In reality, we have ignored the possible contribution of translational diffusion to the scattering fluctuations. We expect that, on average, rotational diffusion causes faster changes than translational diffusion.

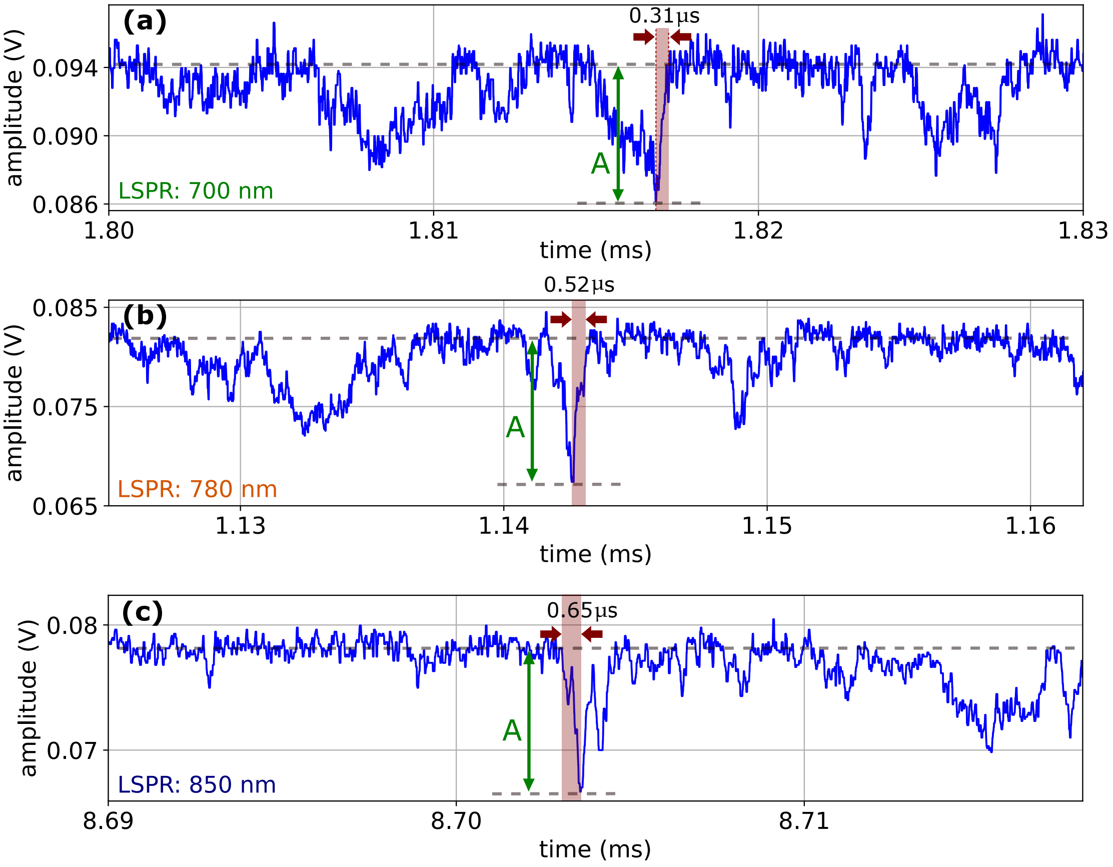

Figure S23: Examples of fast and high amplitude fluctuations of the scattering signal for: **a)** first diffusing-rod sample (15.5 nm length), **b)** second diffusing-rod sample (19.1 nm length) and **c)** third diffusing-rod sample (24.6 nm length). The maximum amplitude of the event is shown as A and the typical times for these fastest changes are indicated.

We consider the amplitude A of the strongest fast sub-burst in each event to estimate the smallest detectable angle. The histograms of max amplitudes (A) are shown in fig. S24 for three samples of diffusing rods. We also consider the noise level N by calculating the standard deviation of the scattering time trace far from any event as $N \approx 0.8\,\text{mV}$.

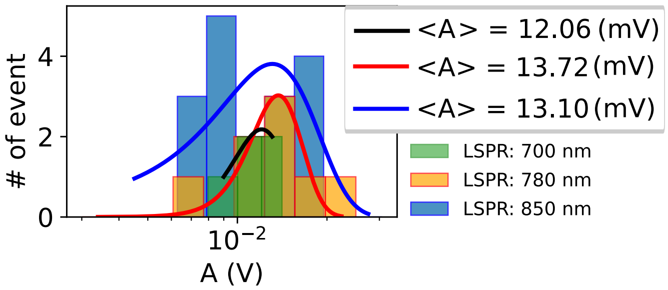

Figure S24: Distribution of maximum amplitude A of the fastest sub-bursts for three samples of diffusing rods: 15.5 nm length diffusing rod (green), 19.1 nm length diffusing rod (orange) and 24.6 nm length diffusing rod (blue). The histograms have been fitted with Gaussian curves with corresponding mean values $\langle A \rangle$. The number of events that have been considered in these histograms are 5, 9 and 19 events respectively for the green, orange and blue histograms.

We estimate the smallest detectable angle by considering the noise level for each sample of diffusing rods as:

$$\delta\theta \approx \frac{90^\circ N}{\langle A \rangle}, \tag{6}$$

which yields: $\delta\theta \approx 5.3^\circ$ for each sample of diffusing rods. The smallest measurable angle is defined for a given sampling rate of our time trace. Our detector, a fast avalanche photodiode (APD, max. bandwidth 400 MHz), is very fast and does not limit the rotational diffusion bandwidth. Its output is digitized at a 50 MHz sampling rate using a fast oscilloscope. The integration time is therefore 20 ns. Therefore, we approximate our sensitivity as:

$$S \approx 5^\circ \sqrt{20\,\mathrm{ns}} = 7 \times 10^{-4}\ deg/\sqrt{Hz}, \tag{7}$$

Note that the sensitivity is proportional to S^{-1}.

Comparing sensitivity with the confocal measurement

Laser focus on the glass

We have measured diffusing gold nanorods in the absence of the sensor GNR in a confocal measurement with linearly polarized excitation and parallel detection. For a typical event

related to the diffusing gold nanord sample with 24.6 nm length diffusing rod, the maximum amplitude is $A \approx 5.5$ mV (see fig. S25) with duration time of $t = 3$ µs to rotate from 0° to 90°.

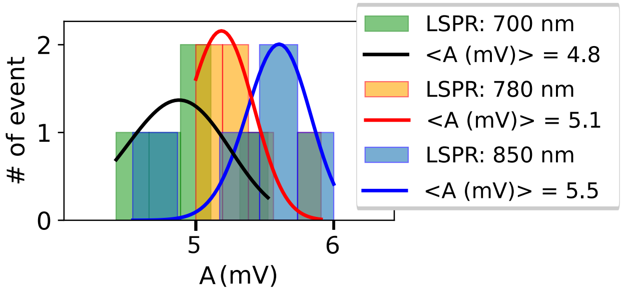

Figure S25: Distribution of maximum amplitude A of the fastest sub-bursts for three samples of diffusing rods. The focus of the laser is on the glass. The histograms in green, orange and blue are respectively for 15.5 , 19.1 and 24.6 nm length diffusing rods. The histograms have been fitted with Gaussian curves with corresponding mean values $\langle A \rangle$. The number of events that have been considered in these histograms are 5, 6, 5 events respectively for green, orange and blue histograms.

We can estimate the smallest detectable angle (by considering the noise level as $N \approx 0.45$ mV) as $\delta\theta \approx \frac{90° N}{A} \approx 7.4°$. Also, the sensitivity is:

$$S \approx 7.4° \sqrt{20\,\mathrm{ns}} = 1 \times 10^{-3}\ deg/\sqrt{Hz}. \tag{8}$$

Therefore, the sensitivity in the near-field method is higher than in the confocal one. As the focus of the laser is on the glass, the detected signal includes both scattered and reflected waves.

Laser focus far from the glass

As a final check, we repeated the measurement 1 µm away from the glass substrate to eliminate the reflected wave. For a typical event in this configuration for the same diffusing nanorod sample (24.6 nm length diffusing rod), the maximum amplitude is $A \approx 3.1$ mV with rise time of $t = 1.5$ µs and the noise level is $N = 0.37$ mV. Therefore, $\delta\theta \approx \frac{90° N}{A} \approx 10.1°$ and

$$S \approx 10.1° \sqrt{20\,\mathrm{ns}} = 1.4 \times 10^{-3}\ deg/\sqrt{Hz}. \tag{9}$$

The angle sensitivity in the dark-field-confocal measurement (1 µm far from the glass) is roughly 1.5 times worse than in on-glass measurement and twice worse than in the near-field measurement.

In conclusion, we compare the angular sensitivities in these three types of scattering experiments: the dark-field scattering of diffusing rods in solution (equation.9), the bright-field scattering measurements (equation.8) and the near-field measurement using a sensor GNR (equation.7). All measurements are done with a sampling time of 20 ns. The incident power in the near-field and confocal measurements are respectively 38 and 160 µW(confocal measurements need higher incident power due to their low signal-to-noise ratio). The power used in the confocal measurement is around 4 times more than the one used in the near-field measurement which affects not only the signal-to-noise ratio but also the sensitivity. Assuming shot-noise-limited detection in the angular-sensitivity comparison, we have to account for a factor of $\sqrt{4} = 2$ in comparing the equations 7, 8 and 9. Consequently, the correction of S by the incident power makes the near-field measurement 3 and 4 times more sensitive in comparison to the bright- and dark-field confocal measurements, respectively.

S9.1 Sensitivity for dielectric diffusing rod in the near-field measurement

According to fig. S1, the amplitude A of the dielectric diffusing nanorod is roughly 500 times smaller than the gold nanorod as diffuser (both with dimension of $22\times5\text{nm}^2$ and with the same sensor GNR $112\times40\text{nm}^2$); therefore, $\delta\theta \approx 5.3° \times 500$. We can estimate the time needed to detect the reorientation of the dielectric diffuser. To do the estimation, first consider a nanorod reorientation from 0° to 90° which needs a time t, $\sqrt{t} = \frac{7\times10^{-4}\ °/\sqrt{Hz}}{90°} \approx$ 60 ps to be detectable. Therefore, the corresponding time for the dielectric nanorod is $\sqrt{t} = \frac{7\times10^{-4}\ °/\sqrt{Hz}\ \times\ 500}{90°} \approx 15\,\mu\text{s}$.

Therefore, to detect reorientation or conformation changes of the dielectric nanorod (such

as proteins), we need a few tens of microsecond.

S9.1.1 Sensitivity expected from simulations for a tethered small nanorod

We have optimized the angular sensitivity by fixing the diffusing-nanorod's ($18\times5\text{nm}^2$) position near the tip of the sensor GNR. To estimate the maximum sensitivity, we calculate the scattering cross section of the sensor-diffusing-rod system with 10 nm surface-to-center distance, i.e., 1 nm metal-to-metal gap. According to fig. S6b, the maximum angle sensitivity occurs around $\theta \approx \pi/8$; therefore, we consider angle changes from $\pi/8$ to $\pi/8 + 1^\circ$ and calculate the change in scattering cross section, $\Delta\sigma_{scat}$=604.8 nm^2. The corresponding signal level can be written as:

$$\frac{\Delta\sigma}{\sigma_0} = 6.3\%. \tag{10}$$

We can estimate the smallest detectable signal by considering the noise level in our near-field measurements from fig. S23 as:

$$\left.\begin{array}{r} N \approx 0.8\,\text{mV} \\ \text{Signal level} \approx 80\,\text{mV} \end{array}\right\} \frac{0.8}{80} = 10^{-2} = 1\%,$$

therefore, the smallest detectable angle in the tethered system is $\frac{1^\circ}{6.3} \approx 0.15^\circ$ (in a time window of 20 ns). The sensitivity parameter can be written as $S = 0.15^\circ\sqrt{20\,\text{ns}} = 2.1 \times 10^{-5}\ deg/\sqrt{Hz}$. By comparing with equation. 7, we find that our near-field measurement with free diffusion is at least 30 times weaker in angle sensitivity than the simulation of an ideal tethered system.

References

(1) Hohenester, U.; Trügler, A. MNPBEM–A Matlab toolbox for the simulation of plasmonic nanoparticles. *Computer Physics Communications* **2012**, *183*, 370–381.

(2) Asgari, N.; Baaske, M. D.; Orrit, M. Burst-by-Burst Measurement of Rotational Diffusion at Nanosecond Resolution Reveals Hot-Brownian Motion and Single-Chain Binding. *ACS nano* **2023**, *17*, 12684–12692.

(3) Larin, K. V.; Akkin, T.; Esenaliev, R. O.; Motamedi, M.; Milner, T. E. Phase-sensitive optical low-coherence reflectometry for the detection of analyte concentrations. *Applied optics* **2004**, *43*, 3408–3414.

(4) Kestin, J.; Sokolov, M.; Wakeham, W. A. Viscosity of liquid water in the range- 8 C to 150 C. *Journal of physical and chemical reference data* **1978**, *7*, 941–948.

(5) Pierres, A.; Benoliel, A.-M.; Zhu, C.; Bongrand, P. Diffusion of microspheres in shear flow near a wall: use to measure binding rates between attached molecules. *Biophysical Journal* **2001**, *81*, 25–42.

(6) Lisicki, M.; Cichocki, B.; Rogers, S. A.; Dhont, J. K.; Lang, P. R. Translational and rotational near-wall diffusion of spherical colloids studied by evanescent wave scattering. *Soft matter* **2014**, *10*, 4312–4323.
